# Shengyu decoction ameliorates knee osteoarthritis by inhibiting endoplasmic reticulum stress via Piezo1 channels

**DOI:** 10.3389/fphar.2025.1592818

**Published:** 2025-07-14

**Authors:** Xuyu Song, Ying Liu, Xianhui Shen, Lei Zhang, Hongwei Kong, Siyi Chen, Lisong Sheng, Rong Sun

**Affiliations:** ^1^ Orthopaedic Trauma Surgery, The Second Hospital of Shandong University, Jinan, Shandong, China; ^2^ Academy of Traditional Chinese Medicine, Tianjin University of Traditional Chinese Medicine, Tianjin, Shandong, China; ^3^ The Second Clinical College, Shandong University, Jinan, Shandong, China; ^4^ Department of Traditional Chinese Medicine, The Second Hospital of Shandong University, Jinan, Shandong, China; ^5^ School of Pharmacy, Shandong University of Traditional Chinese Medicine, Jinan, Shandong, China; ^6^ Shandong Academy of Chinese Medicine, Jinan, Shandong, China; ^7^ Lunan Pharmaceutical Co., Ltd., Linyi, Shandong, China; ^8^ Basic Medical Research Institute, Integrated Traditional Chinese and Western Medicine Center, The Second Hospital of Shandong University, Shandong University, Jinan, Shandong, China; ^9^ Advanced Medical Research Institute, Cheeloo College of Medicine, Shandong University, Jinan, Shandong, China; ^10^ National Administration of Traditional Chinese Medicine, High-level Key Disciplines of Traditional Chinese Medicine, Toxicology of Traditional Chinese Medicine, Cheeloo College of Medicine, Shandong University, Jinan, Shandong, China

**Keywords:** shengyu decoction, knee osteoarthritis, traditional Chinese medicine, endoplasmic reticulum stress, Piezo1

## Abstract

**Background:**

Shengyu decoction (SYD) is a classic and excellent prescription of traditional Chinese medicine (TCM). The innovative use of SYD by Chinese medical master Prof. Qi Shi in the treatment of knee osteoarthritis (KOA) has achieved considerable clinical outcomes. However, the current weakness is the lack of studies on the active ingredients and mechanisms of SYD.

**Purpose:**

To evaluate the role of SYD in reducing KOA cartilage damage as well as to explore the active ingredients and mechanisms of SYD.

**Methods:**

The KOA rat model and chondrocyte model were established. This study employed various molecular biology techniques to clarify the role of SYD *in vivo* and *in vitro*. The active ingredients and mechanisms of SYD were analyzed through ultra-high-performance liquid chromatography–quadrupole time-of-flight mass spectrometry (UPLC-Q-TOF-MS), RNA sequencing, molecular docking, and surface plasmon resonance (SPR). Finally, rescue experiments were conducted to verify the mechanisms.

**Results:**

The results revealed that SYD could significantly reduce cartilage tissue lesions, inhibit inflammation, and regulate proliferation–apoptosis balance. Transcriptome analysis showed an increase in the expressions of Piezo1, Xbp1, and Atf6 in KOA, and SYD downregulated them. UPLC-Q-TOF-MS analysis revealed four bioactive compounds of SYD, which were further confirmed to directly interact with Piezo1 through molecular docking and SPR assays. Furthermore, SYD downregulated the calcium ion concentration and the intensity of Piezo1 and ERS. Meanwhile, in the rescue experiment, Yoda1, the agonist of Piezo1, antagonized the pharmacological effects of SYD.

**Conclusion:**

The present results provide strong evidence that SYD protected articular cartilage via inhibiting the Piezo1-mediated ERS signaling pathway. Overall, our work emphasizes the pivotal role of TCM in addressing medical challenges and provides new ideas for the treatment of KOA.

## Introduction

Knee osteoarthritis (KOA) is a degenerative, chronic joint disease that mainly leads to progressive lesions of cartilage, subchondral bone, and the surrounding synovium (Jang et al., 2021). The most common KOA risk factors are aging, genetic predisposition, and obesity (Giorgino et al., 2023). Nearly 30% of individuals aged >45 years have radiographic manifestations of KOA, and approximately half of them present with knee-related issues (Katz et al., 2021). In general, in the absence of any contraindications, supplementation with topical or oral nonsteroidal anti-inflammatory drugs (NSAIDs) is strongly recommended as the first-line treatment of KOA (Richard et al., 2023). However, non-selective oral NSAIDs have toxicity involving the alimentary, circulatory, and urinary systems. Intra-articular injections of steroids, hyaluronic acids (HA), and platelet-rich plasma (PRP) are also important approaches (Burchard et al., 2019). However, this treatment cannot reverse or repair damaged cartilage. Joint replacement surgery is the only option to improve patients’ quality of life in end-stage KOA when the results of conventional symptomatic treatment are dissatisfactory (Primorac et al., 2020). As a chronic condition with pain as the primary symptom, KOA remains a challenge to treat.

Traditional Chinese medicine (TCM) has a long history of treating KOA. Shengyu decoction (SYD) is a classic and excellent prescription containing seven botanical drugs, namely, *Angelica sinensis* (Oliv.) Diels (*Apiaceae; Angelicae sinensis radix*), Paeonia lactiflora Pall (*Paeoniaceae; Paeoniae radix alba*), *Conioselinum anthriscoides* ‘Chuanxiong’ (*Apiaceae; Chuanxiong rhizoma*), Rehmannia glutinosa (Gaertn.) Libosch. ex DC (*Orobanchaceae; Rehmanniae radix praeparata*), *Bupleurum chinense* DC (*Apiaceae; Bupleuri radix*), *Panax ginseng* C.A.Mey (*Araliaceae; Ginseng radix et rhizoma*), and *Astragalus mongholicus* Bunge (*Fabaceae; Astragali radix*). In TCM theory, SYD focused the whole treatment on qi and blood harmony through “Shaoyang reconciliation,” which yielded remarkable outcomes in KOA treatment.

In clinical trials, Prof. Qi Shi (Yong et al., 2017), a Chinese medical master, and other scholars (Pei-jie et al., 2024) applied SYD to treat KOA and demonstrated remarkable clinical efficacy. Meanwhile, modern pharmacological research has also revealed the potential anti-inflammatory effects of SYD (Zhao et al., 2014). However, there is a lack of reports on basic research; as a result, its substances and mechanisms remain unclear.

Piezo1, a mechanosensitive ion channel expressed by nonsensory tissues, can sense various mechanical loads, including static pressure, shear stress, and membrane stretch (Wang et al., 2020). Piezo-mediated mechanical transduction has been identified as a key regulator connecting the extracellular physical environment with different signal transduction cascades (Lai et al., 2022). Piezo1 is located in the endoplasmic reticulum and cytoplasmic compartments as well as in the nuclear membrane near the cell nucleus (Liu et al., 2022). Due to its inherent mechanical sensitivity, it responds directly to physical forces upon reorganization into lipid bilayers or membrane vesicles. A series of recent studies have demonstrated that the mechanically activated nonspecific cationic channel Piezo1 is involved in many physiological and pathological processes and that it responds to mechanical stress (Cai et al., 2023; Peng et al., 2022). Mechanical injury is a reason for inflammation that runs through the entire disease (Tang et al., 2023). Piezo1 channels act as sensors to convert various mechanical loads to biochemical signals that affect biological processes such as cell proliferation, migration, apoptosis, and vascular remodeling (Shinge et al., 2022). It has been suggested that Piezo1 displayed a positive regulatory role in the inflammation of macrophages (Atcha et al., 2021). In the bone, Piezo1 activates Hippo signaling to enhance the osteogenic effect of mechanical load (Chen et al., 2023a). Li et al. (2019) demonstrated that Piezo1 is a channel through which mechanosensitive ion osteoblasts react to mechanical stress and that it is a new target for treatment. However, the role and the underlying mechanisms of Piezo1 in KOA remain poorly understood.

In this study, we aimed to explore the therapeutic efficiency and mechanisms of SYD on KOA. By constructing *in vivo* and *in vitro* KOA models, KOA-related phenotypes were detected. Furthermore, the components in the drug-containing serum were analyzed through ultra-high-performance liquid chromatography–quadrupole time-of-flight mass spectrometry (UPLC-Q-TOF-MS) technology. RNA sequencing, molecular docking, and surface plasmon resonance (SPR) experiments were then used to predict its potential mechanisms. Finally, rescue experiments were conducted to verify the specific mechanisms through which SYD mediates Piezo1 in the treatment of KOA.

## Materials and methods

### Preparation of SYD

SYD was made up of seven certified commercial Chinese medicinal botanical drugs, namely, *Angelica sinensis* (Oliv.) Diels (*Apiaceae; A. sinensis radix*), *Paeonia lactiflora* Pall (*Paeoniaceae; P. radix alba*), *Conioselinum anthriscoides* ‘Chuanxiong’ (*Apiaceae; C. rhizoma*), *Rehmannia glutinosa* (Gaertn.) Libosch. ex DC (*Orobanchaceae; R. radix praeparata*), *Bupleurum chinense* DC (*Apiaceae; B. radix*), *Panax ginseng* C.A.Mey (*Araliaceae; Ginseng radix et rhizoma*), and *Astragalus mongholicus* Bunge (*Fabaceae; A. radix*). All the botanical drugs were validated taxonomically and checked with the Medicinal Plant Names Services (http://mpns.kew.org/mpns-portal/). The botanical drugs above were purchased from Efang Pharmaceutical Group (Foshan, Guangdong, China, Lot No. A2060271) and passed internal quality control administered by the National Medical Products Administration (China). They were identified by Prof. Qingmei Guo from the Department of Pharmacognosy (Shandong University of Traditional Chinese medicine, China). All botanical drugs were commercially cultivated and processed traditionally according to pharmacopeia standards, with no special processing methods employed. The voucher specimens were deposited in the constant temperature and humidity chamber at our laboratory of the Institute of Basic Medicine, the Second Hospital of Shandong University (Jinan, China).

We adopted the water extraction and alcohol precipitation method for extraction and concentration. The optimized protocol below effectively removes polysaccharides and proteins. The botanical drugs were subjected to water extraction by refluxing with 10 volumes (v/w) of water for 1 h after 30 min of soaking, and this extraction process was repeated twice to ensure exhaustive extraction. Then, combined aqueous extracts were filtered and concentrated to a density of 1 g crude botanical drugs per mL. For ethanol precipitation, pharmaceutical-grade ethanol was gradually added to the concentrated extract until reaching a final concentration of 70% (v/v), followed by overnight storage at 4°C. The supernatant was subsequently collected, and the final extraction yield was calculated to be 2.5%.

### Animals

Thirty-six male Sprague–Dawley (SD) rats (8 weeks old, 280–330 g) were purchased from Beijing Vital River Laboratory Animal Technology Co., Ltd. China (Certificate No. SCXK (Beijing) 2021-0011). The rats were raised at 22°C ± 2°C and 50%–60% humidity under a 12-h light and dark cycle. The rats were allowed free access to standard food and sterile water. Animal studies were approved by the Research Ethics Committee of the Second Hospital of Shandong University (License number: KYLL2024768).

KOA rat models were established with the modified Hulth method (Song et al., 2024). After anesthesia with an intraperitoneal injection of pentobarbital sodium, the rats were placed on the fixed table with supine immobilization, and hair was removed from the left knee. Under sterile conditions, the longitudinal incision on the medial side of the left knee joint was approximately 2 cm in length. The knee joint was dislocated in an extended position and exposed in a flexion position. Then, the medial collateral ligament and anterior cruciate ligament were excised, the medial meniscus was removed, and the joint instability model was established. Based on our prior X-ray evidence, daily forced activity (30 min/day, 4 weeks post-surgery) reliably induced the KOA model (Song et al., 2020). Hence, the same protocol was employed in our study.

The rats were randomly divided into six groups based on a table of random numbers in parallel, including the control group of rats treated only with joint capsule opening and suturing and the model group of rats treated with the modified Hulth method. After calculating based on the administration dosage between humans and rats with the Meeh–Rubner formula, the SYD-L/M/H group of rats received 130.5 mg/kg, 261 mg/kg, or 522 mg/kg extract of SYD. Additionally, based on our previous experience with traditional Chinese herbal compound studies, a 4-week gavage intervention constitutes one treatment cycle. Therefore, tissue samples were collected after 4 weeks of intragastric administration of SYD after KOA modeling (Song et al., 2020). The CXB group of rats received 18 mg/kg CXB (Pfizer Pharmaceuticals Limited, Dalian, China) (Zhao et al., 2025) via intra-articular injection weekly after KOA modeling for 4 weeks (Wu et al., 2024). Rats were sacrificed, and samples were collected at 8 weeks after the surgery.

### Histopathological examination and immunohistochemistry

The specimens of cartilage were fixed in paraformaldehyde and decalcified in EDTA. After dehydration, tissues were fixed in paraffin wax and cut into 10-μm sections. Sections were stained with hematoxylin and eosin (HE) and safranin O-fast green. The Osteoarthritis Research Society International (OARSI) and Mankin scoring system were used to assess the severity of cartilage lesions.

For antigen extraction, slices are treated with citric acid, boiled 5–10 min in a pressure cooker, and then left in 3% H_2_O_2_ for 15 min. The sections were then blocked with goat serum (Servicebio, Wuhan, China) at room temperature for 30 min and incubated with primary antibodies, including aggrecan (Immunoway, YC0042, 1:200), MMP13 (Immunoway, YT2796, 1:200), Col2a1 (Affinity, AF0135, 1:100), Piezo1 (Proteintech, 15,939-1AP, 1:300), ATF6 (Proteintech, 24169-1-AP, 1:100), and XBP1s (Proteintech, 24868-1-AP, 1:300), at 4°C overnight. Then, the slices were incubated with the corresponding secondary antibody at normal temperature for 1 h. Finally, the sections were observed under a digital pathology scanner (NanoZoomer, Shizuoka-ken, Japan).

### Enzyme-linked immunosorbent assay (ELISA)

Blood samples from each group were collected from the heart apex, and the serum was prepared in a centrifuge (3,000 rpm, 15 min, 4°C). IL-1β and TNF-α in serum were measured using a rat IL-1β ELISA Kit (Servicebio, Wuhan, China) and a rat TNF-α ELISA Kit (Servicebio, Wuhan, China), respectively, according to the manual provided by the manufacturers.

### Isolation, culture, and treatment of primary rat chondrocytes

Using the knee joint cartilage of 4-day-old SD rats, trypsin and type II collagenase were used for digestion to obtain primary chondrocytes from rats. Primary chondrocytes were cultured using complete medium (CM) (Gibco, Massachusetts, United States) and intervened according to the following plan. The control group was cultured in ordinary CM, while the model group was cultured in CM with 25 ng/mL IL-1β (Peprotech, Rocky Hill, United States) for 24 h. The SYD-L/M/H group received different concentrations of SYD intervention on the basis of modeling, including 0.625 mg/mL, 1.25 mg/mL, and 2.5 mg/mL. The CXB group was cultured in ordinary CM with 10 μM CXB (Iimoto et al., 2005) (Pfizer Pharmaceuticals Limited, Dalian, China) for 24 h after IL-1β modeling (Wu et al., 2024). In the rescue experiment, the SYD-H + Yoda1 group was combined with 5 μM Yoda1 (Glpbio, Montclair, United States) intervention on the basis of the SYD-H intervention. The SYD-H + Yoda1 + 4-PBA group received an additional 5 mM 4-PBA (Lin et al., 2015) treatment based on the aforementioned interventions.

We used toluidine blue staining to identify whether the cells were chondrocytes. First, the cell culture medium of each group was discarded and washed three times with PBS. Then, the cells were incubated with toluidine blue staining (Servicebio, Wuhan, China) for 2 min and rinsed with PBS immediately. Finally, the cells were observed and photographed under a microscope (200×).

### Western blotting and qPCR

Total protein was collected from each group of cell samples using a Column Tissue and Cell Protein Extraction Kit (Epizyme, Shanghai, China), and the concentration of protein was detected by a BCA Kit (Vazyme, Nanjing, China). The protein samples above were separated on an SDS-PAGE gel after denaturation at 95°C and finally transferred to the PVDF membrane (MilliporeSigma, Burlington, United States) activated by methanol. After being blocked with non-fat milk at room temperature, the membranes were incubated with primary antibodies, including aggrecan (Immunoway, YC0042, 1:1000), MMP13 (Immunoway, YT2796, 1:1,000), Col2a1 (Affinity, AF0135, 1:2000), Piezo1 (Proteintech, 15,939–1-1AP, 1:500), ATF6 (Proteintech, 24169-1-AP, 1:3000), and XBP1s (Proteintech, 24868-1-AP, 1:5000), overnight at 4°C. Then, the membranes were incubated with the corresponding secondary antibody at 4°C for 2 h. Finally, the membranes were detected with a chemiluminescence image analysis system (Tanon, Shanghai, China). The data were analyzed with ImageJ. GAPDH (Proteintech, 10494-1-AP, 1:10000) and β-Tubulin (Proteintech, 10068-1-AP, 1:1,000) were used as loading controls.

The total RNA of each group was extracted using Column Tissue and a Cell RNA Extraction Kit (Vazyme Biotech, Nanjing, China). Then, the cDNA was synthesized with HiScript III RT SuperMix for qPCR Kit (Vazyme Biotech, Nanjing, China). Finally, qPCR was carried out with LightCycler Real-Time system and SuperReal PreMix Plus (Vazyme Biotech, Beijing, China). The primer sequences of relevant genes are in Supplementary Table S1. All target genes were standardized using GAPDH and statistically analyzed using the 2^−△△Ct^ method.

### CCK8, EdU staining, and TUNEL staining

Chondrocytes were inoculated into a 96-well plate and intervened according to the experimental plan. After removing the culture medium of each group, 10 uL CCK8 solution (Vazyme Biotech, Nanjing, China) was added and incubated for 4 h. The absorbance value at 450 nm was detected using an ELISA analyzer (Thermo Fisher, Massachusetts, United States).

Cell viability was detected using EdU staining (Beyotime, Beijing, China). In brief, after the intervention of each group, the culture medium was removed, and 2x EdU reagent was added and incubated for 4 h. Then, 4% paraformaldehyde was added for fixation. Finally, click additive solution and Hoechst 33342 were used and incubated separately for 30 min, followed by observation and photography under a fluorescence microscope (Carl Zeiss, Jena, Germany).

Cell apoptosis was detected using TUNEL staining (Beyotime, Beijing, China). In brief, after using 0.3% Triton-X to intervene in chondrocytes, TUNEL reagent was added and incubated for 60 min at room temperature. Then, the DAPI solution was incubated for 30 min, followed by observation and photography under a fluorescence microscope (Carl Zeiss, Jena, Germany).

### Fluo 3, AM

Fluo-3, AM (Solarbio, Beijing, China), a calcium fluorescent probe, was used for detecting intracellular calcium ion concentration. Each group of chondrocytes was incubated in a 2 μM Fluo-3, AM working solution prepared with DMSO and HBSS for 60 min at 37°C. After removing the above solution, HBSS containing fetal bovine serum was added and incubated for 30 min. Furthermore, the samples were observed and photographed under a confocal laser microscope (Carl Zeiss, Jena, Germany).

### Preparation of drug-containing serum

Six 8-week-old SD rats were fasted and randomly divided into two groups. The rats of the SYD group were given 522 mg/kg/d of SYD by gavage, while the rats of the blank group were free to drink water. After 3 days of intervention, blood was collected and centrifuged to obtain the drug-containing serum for detection.

### UPLC-Q-TOF-MS

The serum above was dissolved in five times its volume of methanol and centrifuged at 12,800 rpm to obtain the supernatant. After repeating the steps three times, the supernatant was retained and diluted with acetonitrile to 10 mg/mL for UPLC-Q-TOF-MS detection. Mass spectrometry detection adopted positive and negative ion scanning modes and adjusted the scan range over 150–1,500. The specific conditions are as follows: spray voltage 3,500(+)/2,800(−), capillary temperature 370°C, and shear gas 50 arb. Liquid chromatograph detection was performed using an ACCUCORE C18 chromatography column (Thermo Fisher, Massachusetts, United States), with acetonitrile A-0.1% formic acid aqueous solution B as the mobile phase. The column temperature was 35°C. The injection volume was 3 μL, and gradient elution was used. The specific parameters are 0–5 min 5%A to 10%A, 5–11 min, 10%A to 28%A, 11–18 min 28%A to 48%A, 18–20 min 48%A to 70%A, and 20–22 min 70%A to 100%A, 0.4 mL/min.

### RNA sequencing and bioinformatics analysis

Cartilage tissues of the control and model groups were selected for RNA sequencing (Agilent 2100 bioanalyzer, Agilent Technologies, CA, United States) with the assistance of Novogene Co., Ltd (Beijing, China). We identified differentially expressed genes with p-value <0.05 and |Log_2_ Fold Change| > 1 as the screening criteria. GO gene functional analysis and KEGG pathway enrichment analysis were performed using the Metascape online platform (http://metascape.org/), and bar charts were drawn for the results.

### Molecular docking and surface plasmon resonance (SPR) analysis

Quercetin, naringin, saikosaponin D, and saikosaponin C were selected from the relevant active ingredients of SYD for molecular docking with Piezo1. The PDB database (https://www1.rcsb.org/) was used to search for the structure of Piezo1, while the PubChem database (https://pubchem.ncbi.nlm.nih.gov/) was used to search for the structure of the active substances. PyMOL 2.3.0 and Chem 3D software programs were used to remove water molecules and optimize molecular mechanics for the optimal conformation of all molecules. Then, Auto Dock Vina v.1.2.0 was used for molecular docking simulations of the target proteins and ligands.

The interaction between compounds and the target protein was quantitatively measured in SPR using the BIACORE T200 system (GE Healthcare Life Sciences, United States). The Piezo1 protein sample (Ipodix, Wuhan, China, PA 2000-5149) was fixed directly onto the carboxymethyl 5 (CM5) sensor chip, and the protein fixation parameters were set to 30 μg/mL, pH 4.5, flow rate 10 μL/min, 25°C. Subsequently, ethylamine was used to block the chip. Different concentrations of small-molecule compounds, including saikosaponin D (RENI, Chengdu, China, TC0175, purity: 99%) and quercetin (RENI, Chengdu, China, TC0717, purity: 99%), were used as analytes, and multi-cycle kinetic detection was employed. The compounds were continuously diluted (0.19–200 μM, 1% DMSO), followed by a flow rate of 30 μL/min for 60 s (binding stage), and then separated at 25°C for 90 s (separation time). The obtained data were analyzed using Biacore T200 software.

### Statistical analyses

The data are reported as mean ± SD (standard deviation) by the SPSS 22.0 software package (San Francisco, United States). One-way analysis of variance (ANOVA) was used to compare three or more groups to assess the significance of differences. The independent-sample T-test was used to compare the differences between the two groups. A p-value <0.05 was considered statistically significant.

## Results

### SYD alleviated cartilage lesions in KOA rats

**FIGURE 1:**
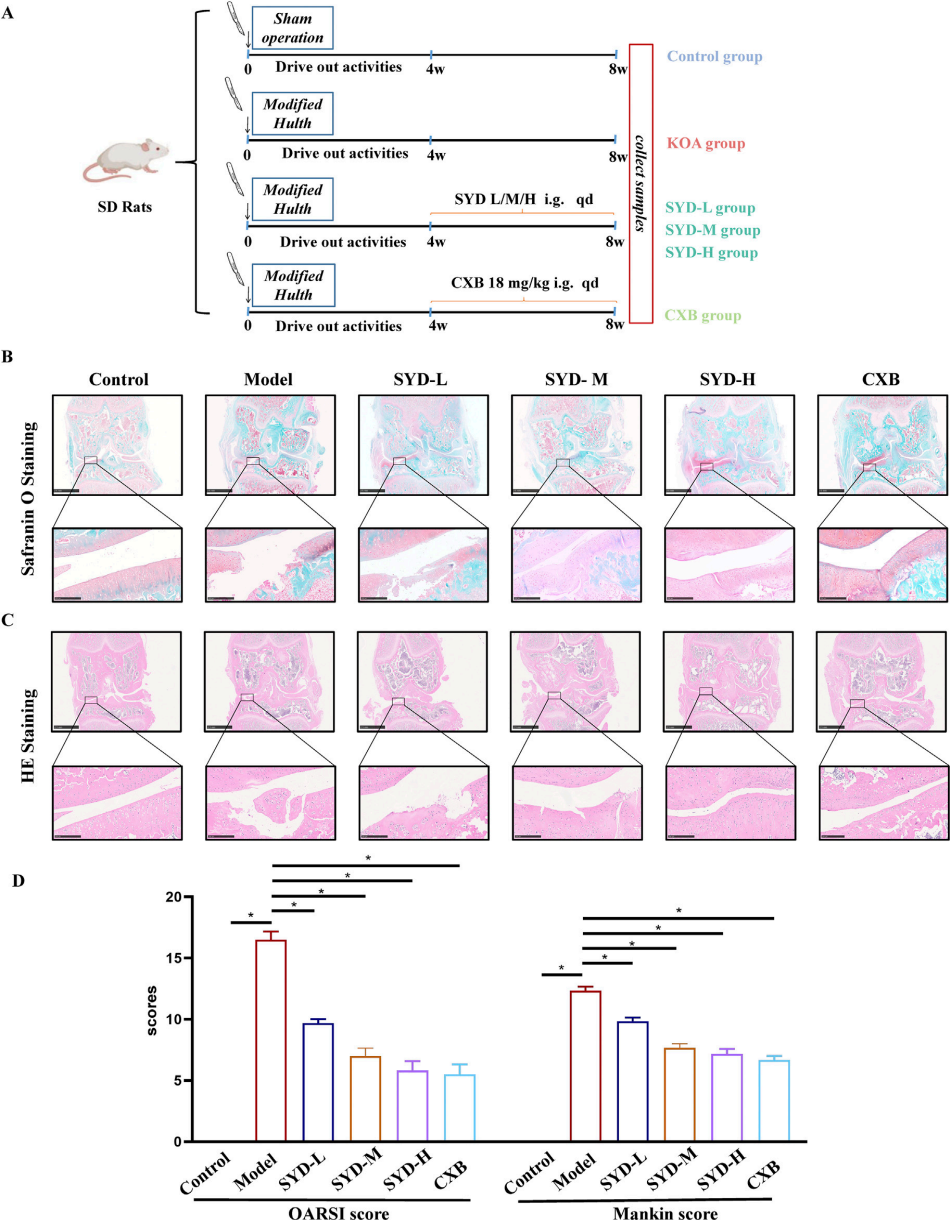
SYD alleviated cartilage lesions in KOA rats. **(A)** Schematic diagram illustrating the animal experiment. **(B, C)** Representative cartilage images of each group with safranin O and HE staining. Scale bar: 2.5 mm and 250 μm. **(D)** Two types of rating quantification for the images shown (N = 3). *p < 0.05.

In order to investigate the efficacy of SYD in ameliorating cartilage lesions on KOA, we first designed appropriate animal experiments. As shown in Figure 1A, the experimental rats were randomly divided into six groups. By adopting the modified Hulth method for KOA modeling and administering different concentrations of SYD by gavage, we used CXB as a positive control. Safranin O-fast green staining and HE staining were performed to evaluate the morphology and structure of cartilage directly, as shown in Figures 1B,C. When compared with the control rats, the model rats showed severe cartilage damage, including erosion, peeling, and huge fissures deep into the subchondral bone, which made it difficult to distinguish the tide line. We found that the joint damage in the three SYD groups was less severe than the damage in the model group. In addition, with an increase in the SYD dose, the surface of the joint cartilage gradually became smoother, with less peeling and shallower cracks, and the tide line became clearer. Notably, the situation of the CXB group was similar to that of the SYD-H group.

The Osteoarthritis Research Society International (OARSI) score and Mankin score were applied to quantitatively evaluate the cartilage damage in each group, and the scores were found to positively correlate with the severity of the lesions. As shown in Figure 1D, the scores of the model group were significantly higher than those of the control group, indicating successful modeling. Meanwhile, the SYD group displayed a dose-dependent decrease in scores (p < 0.05). In addition, the scores of the CXB group were similar to those of the SYD-H group (p > 0.05).

### SYD inhibited inflammation and cartilage degeneration in KOA rats

**FIGURE 2:**
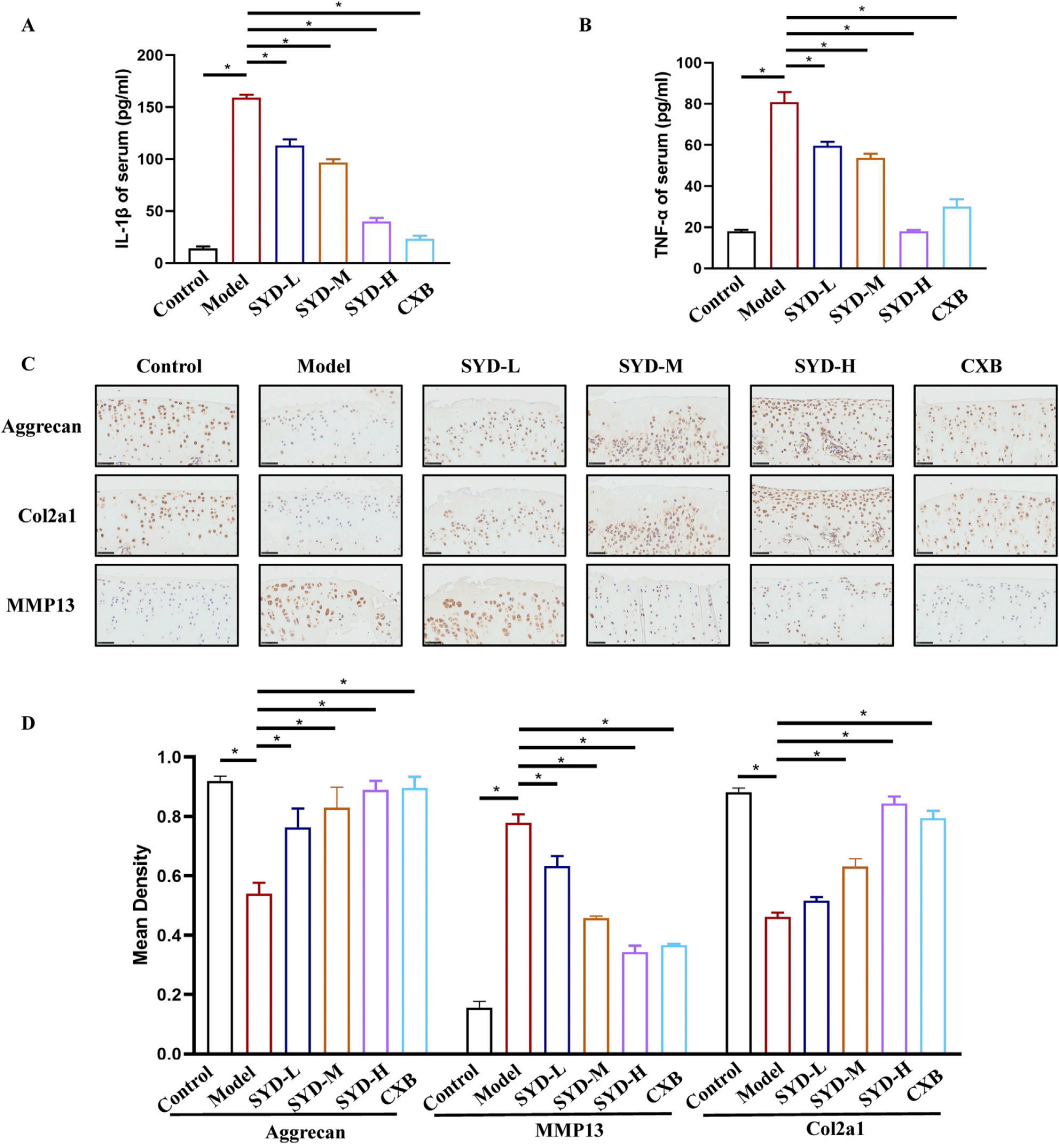
SYD inhibited inflammation and cartilage degeneration in KOA rats. **(A,B)** The levels of IL-1β and TNF-α in serum (N = 3). **(C)** IHC staining for proteins related to cartilage degeneration in the cartilage of different groups. Scale bar: 50 μm. *p < 0.05 **(D)** The quantitative analysis of IHC.

The levels of IL-1β and TNF-α in the serum were induced in response to the inflammatory state of the body and measured by ELISA to reflect the anti-inflammatory effect of SYD (Figures 2A,B). When compared with the control group, the expressions of IL-1β and TNF-α in the model group were significantly increased, which indicated a severe inflammatory response during the KOA process. As the dosage of SYD increased, the expressions of IL-1β and TNF-α gradually decreased, which indicated a dose-dependent inhibition of the inflammatory response by SYD. Notably, CXB had the best effect in reducing IL-1β, albeit the downregulation of TNF-α was not as effective as that of SYD-H.

Immunohistochemistry (IHC) was carried out to detect the changes in proteins in relation to cartilage degeneration. In Figure 2C, when compared to the control group, the expressions of aggrecan and Col2a1 in the model group were significantly downregulated, while the expression of MMP13 was increased, indicating severe cartilage degeneration in this group. After the SYD intervention, the expressions of aggrecan and Col2a1 became positively correlated with the dosage of SYD, while the opposite was true for MMP13. Thus, the situation of the CXB group seemed similar to that of the SYD-H group. The quantitative analysis of IHC is shown in Figure 2D.

### SYD promoted proliferation while inhibiting apoptosis *in vitro*

**FIGURE 3:**
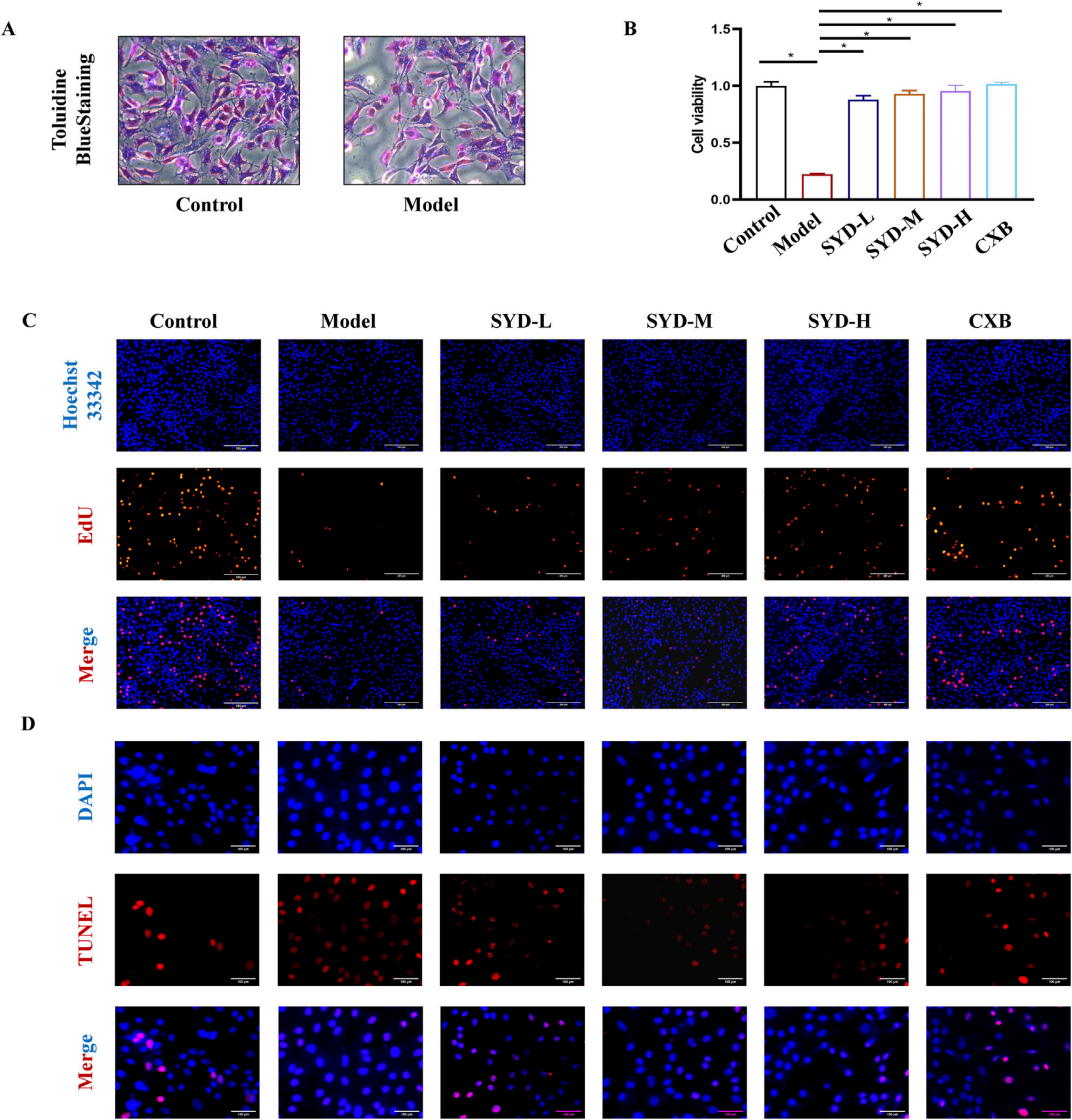
SYD promoted cell viability and proliferation *in vitro*. **(A)** Identification of chondrocytes with toluidine blue staining (200×). **(B)** Cell viability detection with CCK8 (N = 4). **(C)** Cell proliferation detection with EdU staining. Scale bar: 200 μm. *p < 0.05. **(D)** TUNEL staining for detecting the levels of apoptosis. Scale bar: 100 μm.

We first identified the isolated primary cells (Figure 3A), which had a polygonal or spindle-shaped morphology and were stained purple-red with toluidine blue. The cell morphology of the model group was consistent with that of the control group, albeit the density was lower in the former. As such, all the cells were identified as rat chondrocytes.

Figures 3B,C show the results of the CCK8 assay and EdU staining performed to explore cell viability and proliferation, respectively. When compared with the control group after the IL-1β modeling intervention, cell viability and the number of positive cells labeled with EdU significantly decreased. As the dosage of SYD increased, cell viability and the number of proliferating cells also increased significantly. Simultaneously, CXB demonstrated excellent cell proliferation-promoting effects in the CCK8 assay and EdU staining.

Notably, chondrocyte apoptosis was an important part of cartilage degeneration; therefore, TUNEL staining was carried out to detect the degree of apoptosis (Figure 3D). The marked feature of the model group was a significant increase in the labeled apoptotic cells. After an intervention with SYD, apoptotic cells displayed a dose-dependent decrease.

### SYD inhibited cartilage degeneration *in vitro*

**FIGURE 4:**
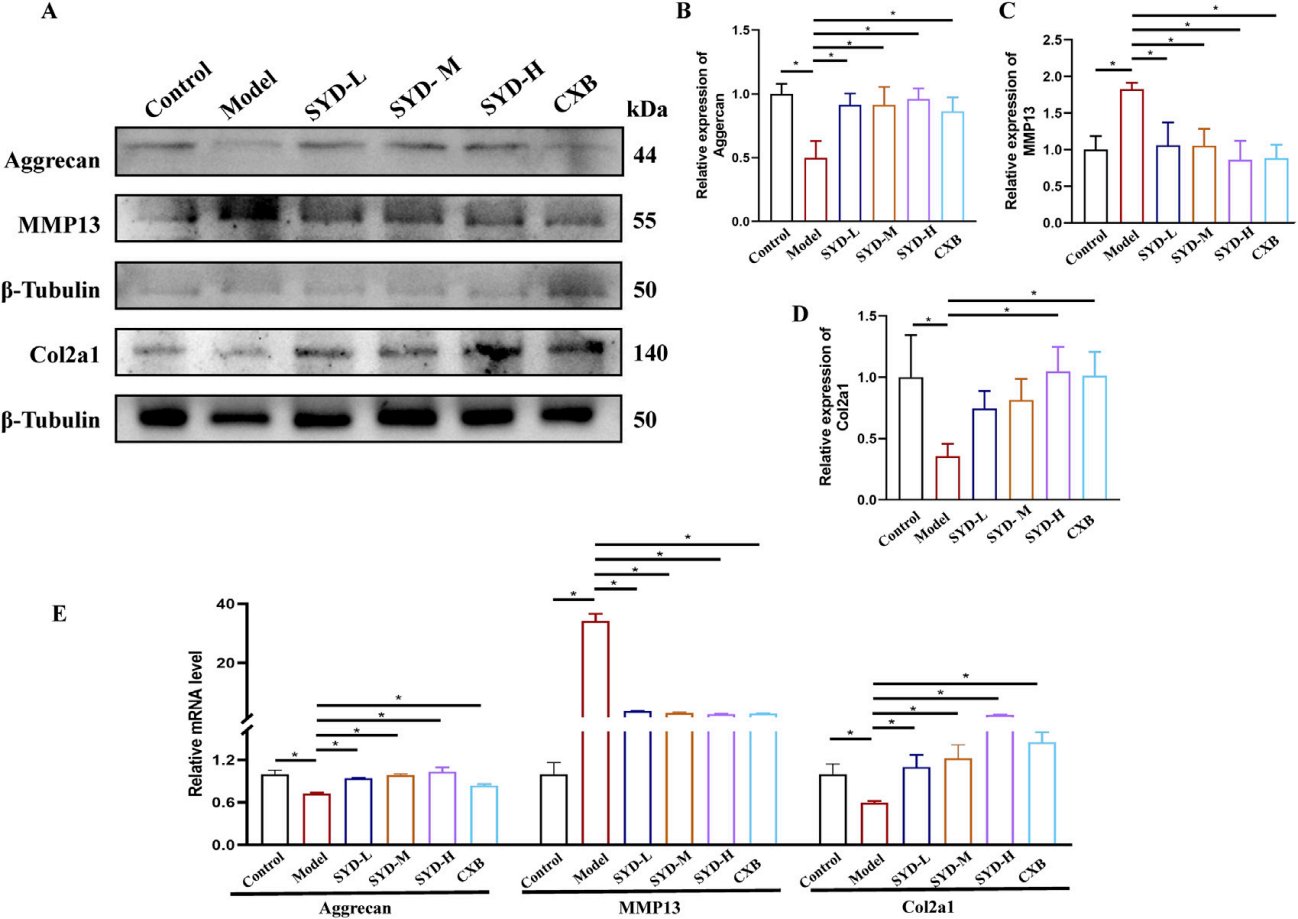
SYD inhibited apoptosis and cartilage degeneration *in vitro*. **(A–D)** Representative blot images and quantitative analysis of aggrecan, MMP13, and Col2a1 in Western blotting (N = 3). **(E)** The mRNA expressions of aggrecan, MMP13, and Col2a1 were detected by qPCR. (N = 3–4) *p < 0.05.

The levels of genes related to cartilage degeneration displayed a protective effect of SYD *in vitro*, as shown in Figures 4A–D. Western blotting analysis revealed that the expressions of the model group of Aggrecan and Col2a1 were significantly downregulated when compared to that in the control group, while the level of MMP13 was significantly upregulated. SYD treatment significantly increased aggrecan and Col2a1 expressions and downregulated the MMP13 expression, both indicating a dose-dependent effect. At the same time, the effect of CXB was similar to that of SYD-H. qPCR detection of the mRNA expressions of the abovementioned genes confirmed the results of Western blotting (Figure 4E).

### Piezo1-regulated Ca^2+^ concentration and ERS may play a key role in KOA and SYD

**FIGURE 5:**
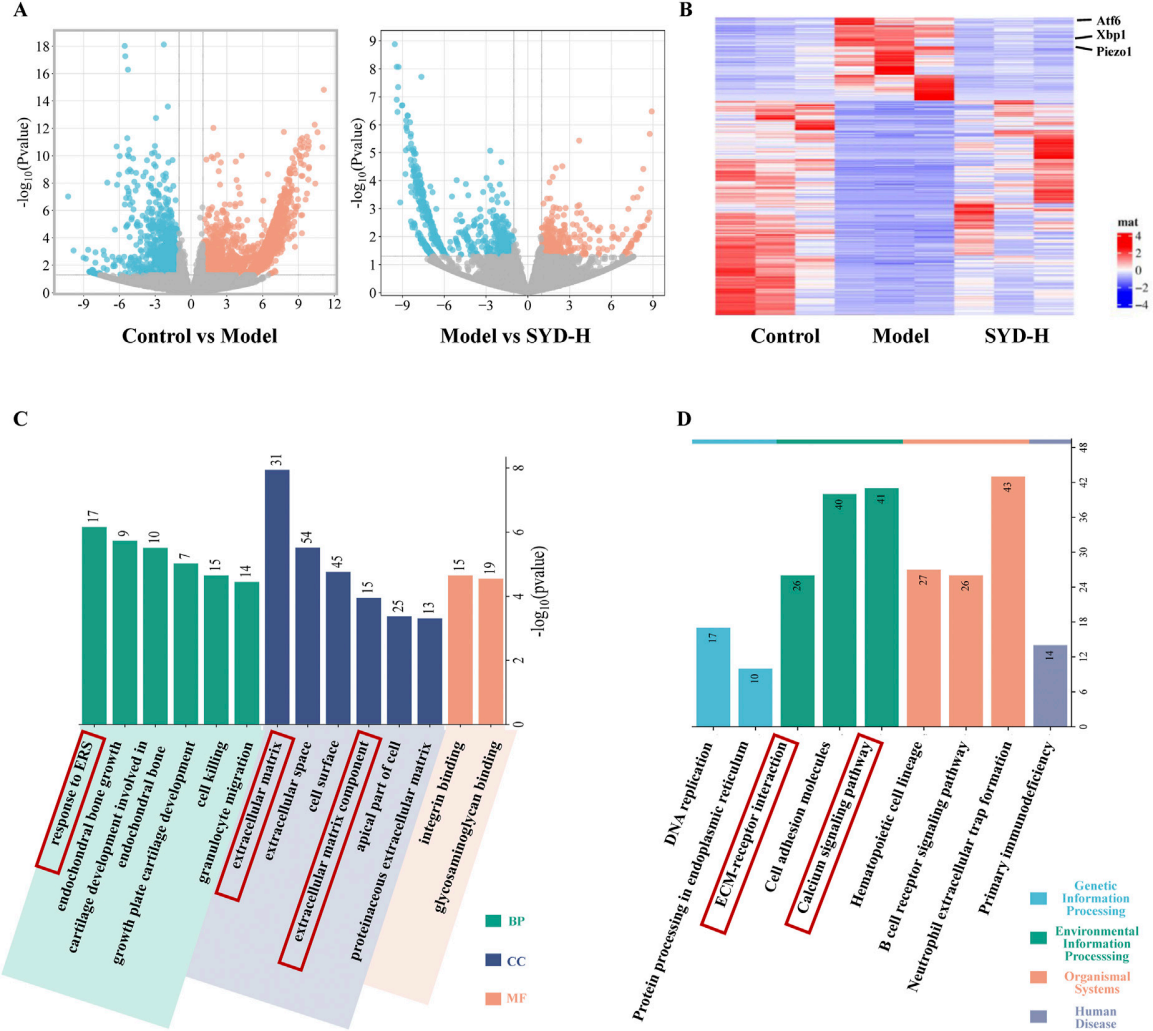
The results of RNA sequencing (Control, Model, and SYD-H). **(A,B)** The differential gene expression among control, model, and SYD-H groups (volcano plot and heatmap, N = 3). **(C)** The results of GO enrichment analysis, including CC, MF, and BP. **(D)** The results of the KEGG pathway enrichment analysis.

RNA sequencing was performed to reveal the changes among the control, model, and SYD-H group in depth. We identified 2,944 differentially expressed genes (DEGs) in the control and model groups and 930 DEGs in the model and SSD-H groups with p-value <0.05 and |Log_2_ Fold Change| >1 as the screening criteria. A volcano plot and heat map were created (Figures 5A,B). The heatmap shows significant changes in the expression of ATF6, Xbp1, and Piezo1 among the three groups (Figure 5B). Subsequently, GO and KEGG enrichment analyses were conducted based on the results of DEGs (Figures 5C,D). What interests us is that the GO analysis showed that SYD affected ERS and the extracellular matrix, while the KEGG analysis showed that SYD regulated the calcium signaling pathway. These results strongly sparked our interest, and we decided to focus on the in-depth exploration of calcium ion regulation and ERS in our subsequent studies.

### Identification of the active ingredients of SYD and verification of predicted targets by molecular docking and SPR

**FIGURE 6:**
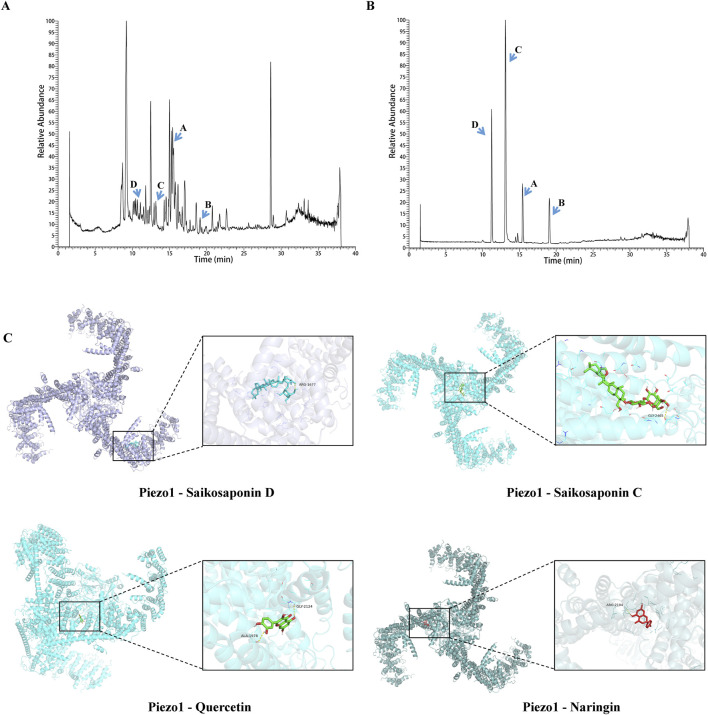
Identification of the key active ingredients of SYD and molecular docking. **(A)** The TIC in the ESI-mode of the SYD extract by UPLC-Q-TOF-MS. **(B)** The chromatographic peaks of saikosaponin C, saikosaponin D, quercetin, and naringin with reference substances in the ESI chromatogram. **(C)** The molecular docking results with Piezo1.

In order to control quality and investigate the active ingredients of SYD, UPLC-Q-TOF-MS was performed to detect the SYD extract and its drug-containing serum in rats. We obtained the total ion chromatogram (TIC) of SYD extract in the negative (ESI−) ionization modes, as shown in Figure 6A, and the positive (ESI+) modes are shown in Supplementary Figure S1A. Supplementary Figures S1C, D show the TIC of SYD drug-containing serum in rats. On comparing with the relevant databases, we identified the top 200 chemical compositions in the two modes mentioned above (Supplementary Tables S2 and S3). Then, we detected some reference substances and identified them as the SYD extract in TIC (Figures 6A, B; Supplementary Figure S1A, B). We found the concentration and content of four compounds—saikosaponin C, saikosaponin D, quercetin, and naringin—are high in SYD, as shown in Table 1. The four active ingredients were subjected to molecular docking with Piezo1, and all binding energies were consistently below −7.5, as shown in Figure 6C.

**TABLE 1:** The concentration and content of four compounds in SYD.

Compound	RT (min)	Theoretical MW	Observed MW	δppm	Concentration (μg/mL)	Content (mg/g)
Saikosaponin C	15.41	925.51553	925.51196	3.86	3.85731	0.33542
Saikosaponin D	19.04	779.45762	779.45673	1.14	1.14182	0.09929
Quercetin	13.14	301.03428	301.03522	3.12	3.12257	0.27153
Naringin	11.28	579.17083	579.17224	2.43	2.43451	0.2117

It is noteworthy that although these four compounds are highly abundant in SYD, quercetin and naringin’s structural characteristics predispose them to bind with multiple proteins, potentially generating false-positive results that classify them as pan-assay interference compounds (PAINS) (Baell and Holloway, 2010). To validate the results of molecular docking, we employed SPR technology for verification, as it can effectively characterize the interactions between proteins and small-molecule compounds. The results showed that both saikosaponin D and quercetin could bind to Piezo1, and the kinetic test curves showing the binding of Piezo1 to saikosaponin D and quercetin are presented in Supplementary Figure S3.

### SYD regulated Ca^2+^ concentration and ERS *in vitro* and *in vivo*

**FIGURE 7:**
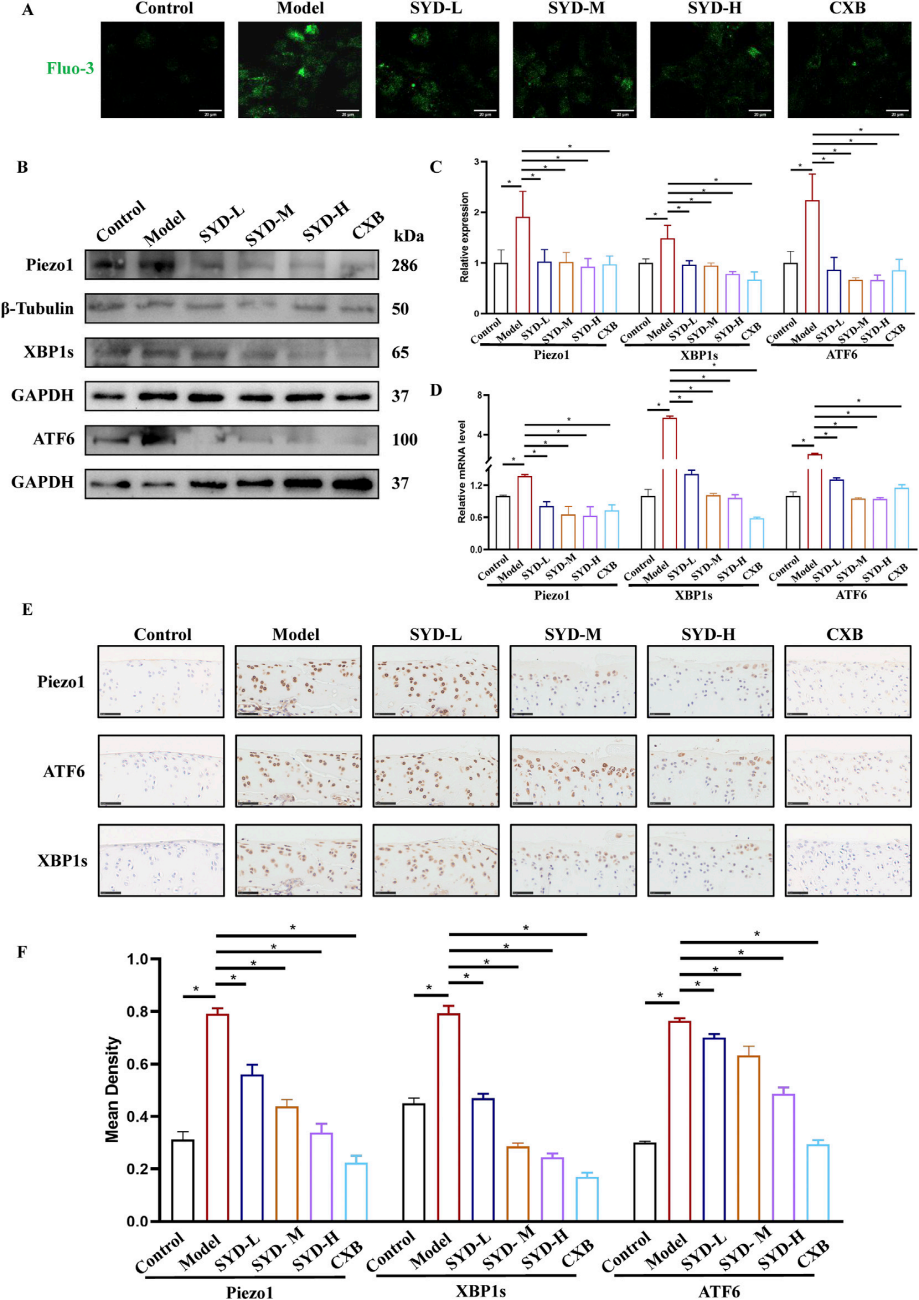
SYD regulated Ca^2+^ concentration and ERS *in vitro* and *in vivo*. **(A)** The detection of Ca^2+^ concentration by Fluo-3,AM. Scale bar: 20 μm. **(B,C)** Representative blot images and quantitative analysis of proteins related to ERS, including Piezo1, XBP1s, and ATF6, in Western blotting. (N = 3) **(D)** The mRNA expressions of Piezo1, XBP1s, and ATF6 were detected by qPCR. (N = 3–4) **(E)** IHC staining for proteins related to ERS in the cartilages of different groups. Scale bar: 50 μm *p < 0.05 **(F)** The quantitative analysis of IHC.

A Ca^2+^ probe, Fluo-3,AM, was used to measure the intracellular calcium concentration in chondrocytes under laser scanning confocal microscopy (LSCM). As shown in Figure 7A, the results indicated that the intracellular calcium concentration increased after modeling intervention but decreased to varying degrees after SYD administration. The calcium concentration in the CXB group appeared similar to that in the SYD group. We performed Western blotting and qPCR to measure the levels of Piezo1 and ERS *in vitro* (Figures 7B–D). The expressions of Piezo1 and ERS-related genes, namely, XBP1s and ATF6, in the model group were significantly increased when compared to that in the control group, while the expression of the aforementioned three genes displayed a significant dose-dependent decrease after SYD intervention. Similarly, CXB can reduce their expression when compared to that in the model group. These results are consistent in terms of the transcription and translation levels.

In Figure 7E, IHC staining of cartilage tissue confirmed the levels of Piezo1 and ERS *in vivo*. In the model group, the numbers of positive chondrocytes for Piezo1, XBP1s, and ATF6 were significantly increased when compared to those in the control group. The number of positive cells for the abovementioned three genes in the SYD-L/M/H groups decreased to varying degrees, seemingly negatively correlated with the dosage. The situation in the CXB group appeared to be similar to that of SYD-H. The quantitative analysis of IHC in Figure 7F also confirmed the above results.

### SYD protected cartilage through the piezo1-regulated ERS signaling pathway in KOA treatment through rescue experiments

**FIGURE 8:**
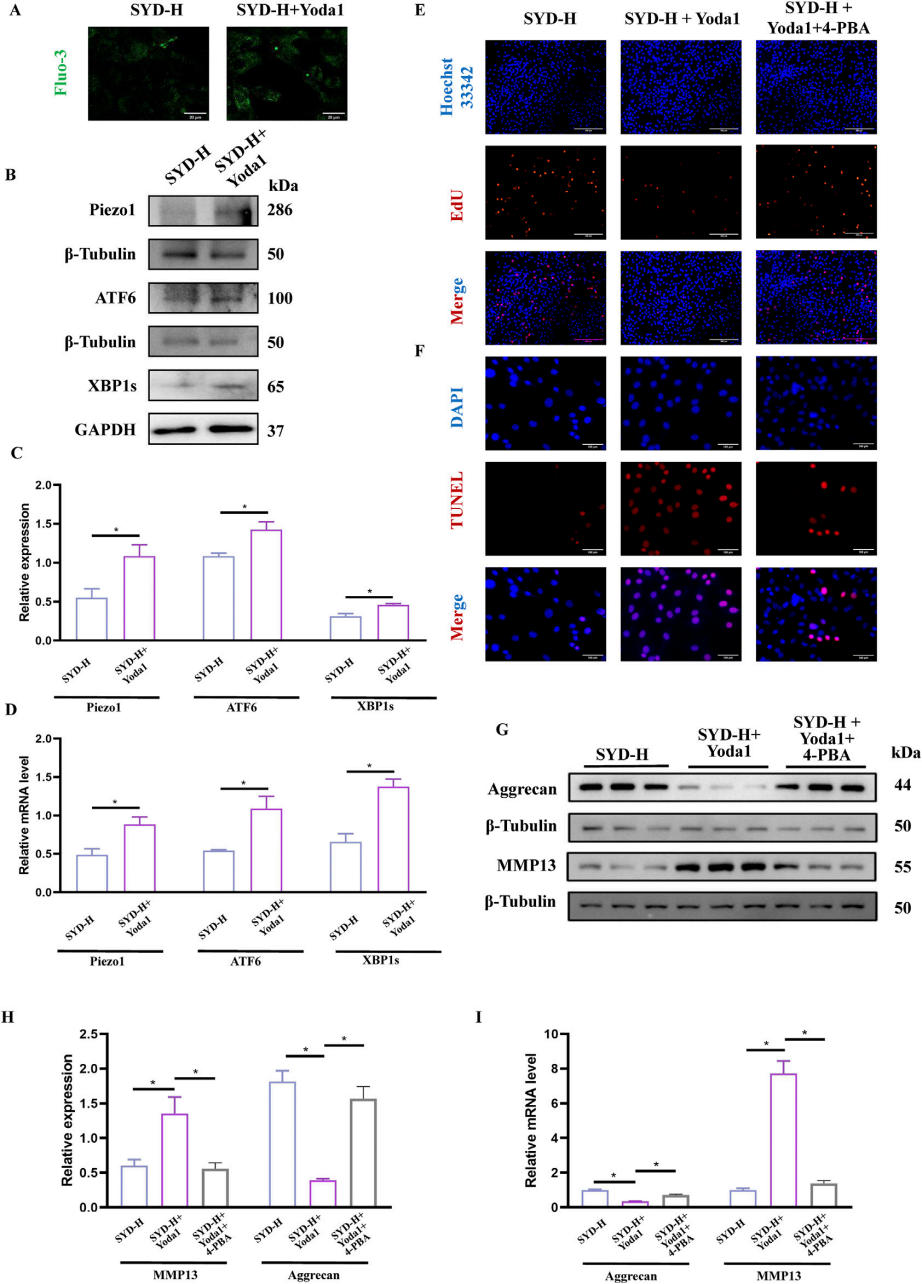
SYD-protected cartilage through Piezo1-regulated ERS signaling pathway by rescue experiments. **(A)** The detection of Ca^2+^ concentration by Fluo-3,AM. Scale bar: 20 μm. **(B,C)** Representative blot images and quantitative analysis of Piezo1 and ERS, including Piezo1, XBP1s, and ATF6, in Western blotting (N = 3) **(D)** The mRNA expressions of Piezo1, XBP1s, and ATF6 detected by qPCR (N = 4). **(E,F)** The EdU (Scale bar: 200 μm) and TUNEL staining (Scale bar: 100 μm) for the detection of proliferation and apoptosis. **(G,H)** Representative blot images and quantitative analysis of proteins related to cartilage degeneration, including aggrecan and MMP13, in Western blotting (N = 3). **(I)** The mRNA expressions of aggrecan and MMP13 were detected by qPCR (N = 4). *p < 0.05.

Based on the experimental results, we chose Yoda1, the selective Piezo1 agonist, and 4-PBA, the ERS inhibitor, to conduct rescue experiments in combination with SYD-H. In Figure 8A, we intuitively observed that the fluorescence signal significantly increased after the Yoda1 intervention, indicating an increase in the Ca^2+^ concentration. Both Western blotting and qPCR results showed that Yoda1 could significantly upregulate the levels of Piezo1, ATF6, and XBP1s, as shown in Figures 8B–D, suggesting that Yoda1 enhanced the expression of Piezo1 and the intensity of ERS.

EdU and TUNEL staining were performed to display the proliferation and apoptosis of chondrocytes (Figures 8E,F). When Yoda1 was used in combination, the number of EdU stain-positive cells decreased while the number of TUNEL stain-positive cells increased. However, the addition of 4-PBA produced paradoxical effects. The protein and mRNA levels of aggrecan and MMP13—the genes related to cartilage degradation—were also detected. As shown in Figures 8G–I, the expression of aggrecan was significantly downregulated while the level of MMP13 was clearly enhanced in the SYD-H + Yoda1 group, thereby illustrating that Yoda1 antagonized the inhibitory effect of SYD on cartilage degradation. Notably, the addition of 4-PBA reversed the expression patterns of aggrecan and MMP13.

## Discussion

KOA is a common orthopedic disease, and according to the relevant epidemiological research reports, there are approximately 300 million patients with KOA across the world (Katz et al., 2021). Although it is not fatal, it involves strong concealment in the early stages and a high disability rate in the later stages; moreover, it has been listed as the 11th most disabling disease in the world (Giorgino et al., 2023). The treatment methods for KOA in modern medicine can be categorized as conservative or surgical treatment. Among the options, oral or topical NSAIDs have been listed as the first-line recommended drugs in KOA treatment guidelines (Katz et al., 2021; Sharma, 2021), albeit long-term use can easily induce gastrointestinal side effects and increase the risk of cardiovascular accidents (Quicke et al., 2022). The high cost of surgical procedures, potential surgical risks, postoperative functional recovery, and postoperative revision continue to pose significant challenges (Jang et al., 2021). When faced with these challenges in treatment, it is clear that finding safer and more effective treatment plans is an urgent scientific problem that must be resolved in the field of KOA research.

TCM has a long history of treating KOA with significant therapeutic effects, and it has been recommended for use in multiple treatment guidelines. SYD is a classic formula derived from *“Yi Zong Jin Jian (The Golden Mirror of Medicine)”* volume 62, and it consists of the root of *Radix Bupleuri* (Chai Hu), the root of *Panax ginseng C.A. Mey* (Ren Shen), and the root of *Astragalus membranaeus (Fisch.) Bge* (Huang Qi) added to the Siwu decoction. Interestingly, the Chinese medical master Prof. Qi Shi and other scholars applied the theory of “Shaoyang being in charge of bone” originating from “*The Huangdi Neijing,”* also known as *“The Inner Canon of Huangdi”* or *“Yellow Emperor’s Inner Canon,”* for using SYD to treat KOA, indicating Shaoyang reconciliation, to achieve remarkable outcomes in the clinical setting (Pei-jie et al., 2024; Yong et al., 2017). However, there is still a lack of basic research on the treatment of KOA with SYD. The active substances and the mechanisms of SYD continue to remain unclear. Therefore, in the present study, we focused on the abovementioned issues in order to contribute to the wisdom of TCM in the treatment of KOA.

**FIGURE 9:**
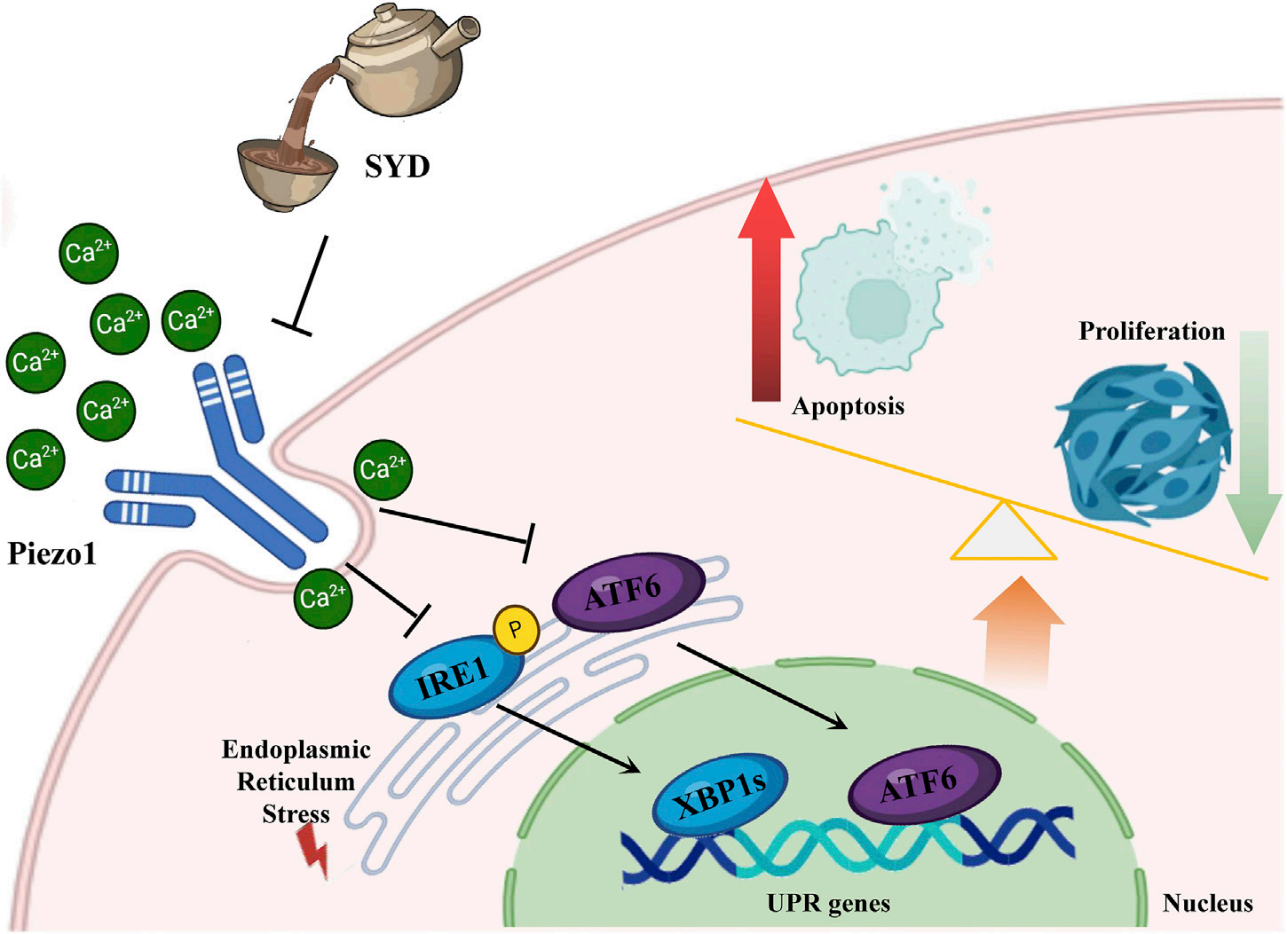
Depiction of the mechanism diagram of SYD in reducing cartilage damage.

The overall protocol design of this work is shown in Figure 9. A confirmed therapeutic effect is a prerequisite for subsequent research; therefore, we first detected the efficacy of SYD *in vivo* (Figures 1, 2). Safranin O-fast green and He staining facilitated the visualization of cartilage damage, suggesting that the severely damaged cartilage after modeling was significantly reduced after SYD intervention and that the effect was dose-dependent. When combined with the subsequent ELISA and IHC staining results, it was confirmed that SYD inhibited cartilage degeneration and inflammation and significantly reduced cartilage damage; its efficacy is thus reliable, which provides a guarantee for subsequent work. As shown in Figures 3, 4, we conducted a deeper study on the phenotype of SYD *in vitro*. The results showed that SYD enhanced the viability of chondrocytes, promoted proliferation, inhibited apoptosis, and regulated the expression of cartilage degradation-related genes in a dose-dependent manner. These results were consistent with the animal experimental results, indicating the reliable effect of SYD *in vivo* and *in vitro*.

**FIGURE 10:**
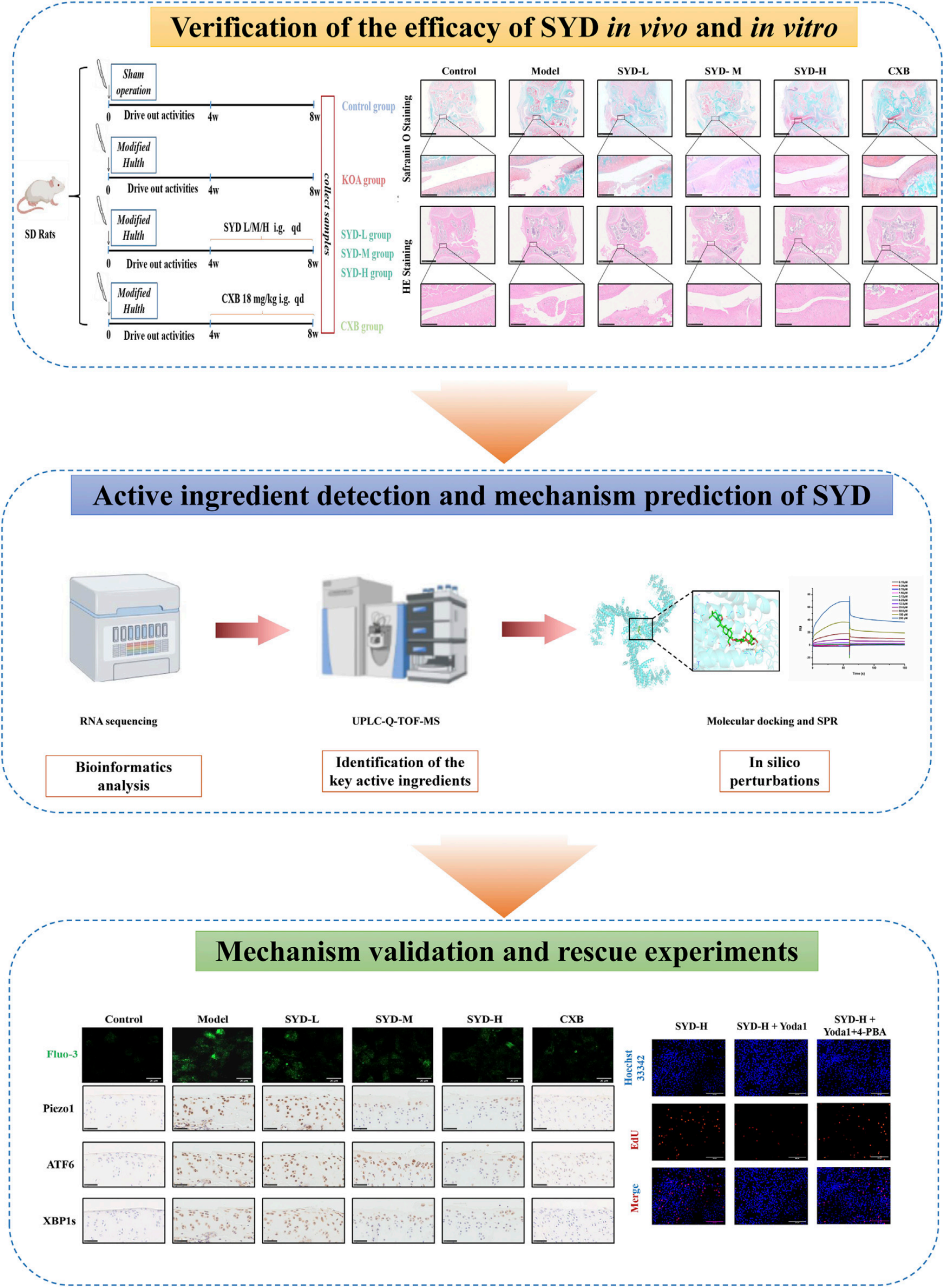
Overall protocol design of this work.

Transcriptomic analysis (Figures 5A–D) showed a significant upregulation of the expressions of Piezo1, Xbp1, and Atf6 in KOA and a downregulation of these genes in SYD-H. GO and KEGG analyses were performed to analyze the functions and enrichment pathways of genes, respectively, so as to provide important clues. Based on the above results, the ERS and calcium ion signaling pathways sparked our research interest.

The identification of active ingredients in SYD can elucidate its pharmacological activity. Therefore, we identified four major components in SYD with relatively high content using UPLC-Q-TOF-MS, namely, saikosaponin C, saikosaponin D, quercetin, and naringin (Figures 6A,B). Molecular docking demonstrated their strong binding affinity with Piezo1. However, it is noteworthy that quercetin and naringin may exhibit non-specific binding to multiple protein targets, potentially leading to false-positive results, and are therefore generally classified as PAINS (Baell and Holloway, 2010). Research has shown that while PAINS exhibit widespread activity *in vitro*, their action mechanisms are often nonspecific (Baell and Walters, 2014; Dahlin et al., 2015). Thus, molecular docking outcomes are essentially predictive.

The TCM compound prescriptions do contain some PAINS, but this will not affect their efficacy and mechanism. Potential factors affecting the efficacy of TCM compound prescriptions may be associated with multi-component synergistic effects and absorption-enhancing mechanisms. Specifically, the self-assembly phenomenon of polyphenols, saponins, or other bioactive compounds may generate multi-component synergistic effects, thereby enhancing the therapeutic efficacy (Guo et al., 2024). Meanwhile, the multi-component compatibility can promote absorption and enhance bioavailability (Zhu et al., 2013). Therefore, despite the presence of PAINS in SYD, it still exhibited good activity in our study.

We conducted SPR experiments to further validate the binding capability with Piezo1, and the results also confirmed their binding capability (Supplementary Figure S3). Piezo1 is composed of three structures resembling curved leaves that surround a small hole. When the cells are subjected to mechanical stimulation, the leaf structure responds to the stimulation and becomes deformed, and the mechanical signal activates the ion channel through the Piezo1 protein pore nucleus structure, resulting in Ca^2+^ influx (Wang et al., 2022). Piezo1 serves as an important target in regulating KOA progression and participates in the mechanical signal transduction process of chondrocytes (Gao et al., 2022; Lai et al., 2022). It converts mechanical signals to chemical signals, thereby regulating a series of biological effects of chondrocytes (Lee et al., 2021; Quicke et al., 2022).

Under the stimulation of changes in the calcium ion concentration or internal and external environment, the number of unfolded proteins also increases. When this accumulation exceeds the ER’s folding and processing capacity, the unfolded protein response (UPR) triggers the ERS to restore protein homeostasis or, under prolonged or excessive stress, to initiate apoptosis and other forms of programmed cell death (Chen and Cubillos-Ruiz, 2021). The UPR is primarily mediated by three canonical signaling pathways, including the PERK–eIF2α–ATF4 pathway, the IRE1–XBP1 pathway, and the ATF6 pathway. And all three UPR branches can be co-activated (Lu et al., 2024).

Ca^2+^ functions as both the principal calcium store within the ER and a critical second messenger in cellular signaling (Chen et al., 2023b). Cytoplasmic Ca^2+^ overload further exacerbates ERS by activating CaMKII and promoting the opening of L-type calcium channels (LTCCs), creating a positive feedback loop that sustains UPR signaling. The molecular basis of ERS sensing and Ca^2+^ homeostatic feedback has also been clarified (Wang et al., 2023).

Related studies have confirmed that excessive or persistent ERS in KOA chondrocytes can increase apoptosis and reduce protective responses, thereby exacerbating KOA (Tan et al., 2020).

Therefore, it is urgent to verify whether SYD regulates calcium ion concentration through Piezo1 so as to inhibit ERS and exert a protective effect on cartilage. After intervention with SYD (Figure 7), the Ca^2+^ concentration in chondrocytes significantly decreased, as indicated by the decrease in Piezo1 expression both *in vitro* and *in vivo*. Similarly, ERS resulted in varying degrees of inhibition after SYD intervention. We conducted a rescue experiment using Yoda1 and 4-PBA, the selective agonist of Piezo1 and inhibitor of ERS, as illustrated in Figure 8. Through analysis of the results, it was imperative that the application of Yoda1 significantly increased the expression of Piezo1 and the concentration of Ca^2+^ while also enhancing ERS. The part of the study on KOA-related phenotypes found that Yoda1 antagonizes the protective effect of SYD, specifically reducing cell proliferation, promoting apoptosis, and accelerating cartilage degeneration. However, significant administration of 4-PBA antagonized these effects. And the overall mechanism diagram and protocol design of this work were shown in Figures 9, 10.

Taken together, the present results provide a scientific basis for the treatment of KOA with TCM and provide insight toward promoting the inheritance, innovation, and development of TCM.

## Conclusion

Through the present results, we confirmed the definite role of SYD in protecting cartilage, which includes inhibiting inflammation and apoptosis, promoting proliferation, and regulating gene-related cartilage degeneration. Subsequently, the UPLC-Q-TOF-MS results of the drug-containing serum revealed the active ingredients of SYD and predicted the mechanisms of SYD when combined with RNA sequencing. The results of the rescue experiments confirmed that SYD inhibited the Piezo1-regulated ERS signaling pathway to protect cartilage. Overall, our work focused on the modernization study of TCM and emphasized the significant role of TCM in KOA treatment.

## Data Availability

The data presented in the study are deposited in the GEO repository (https://www.ncbi.nlm.nih.gov/geo/), accession number GSE301460.

## Abbreviations

CXB, celecoxib; EIC, extracted ion chromatograms; ERS, endoplasmic reticulum stress; GO, Gene ontology; KEGG, Kyoto Encyclopedia of Genes and Genomes; KOA, knee osteoarthritis; OARSI, The Osteoarthritis Research Society International; SYD, Shengyu decoction; TCM, traditional Chinese medicine; UPLC-Q-TOF-MS, ultra-high-performance liquid chromatography-quadrupole time-of-flight mass spectrometry.
