# Supplementary material for: Shengyu decoction ameliorates knee osteoarthritis by inhibiting endoplasmic reticulum stress via Piezo1 channels
Source: Front Pharmacol. 2025 Jul 14;16:1592818. doi: 10.3389/fphar.2025.1592818 (PMC12301362; doi:10.3389/fphar.2025.1592818)
Supplement: Supplementary file 1 [file Table1.docx]

**Supplementary Table 1**

| **Gene** | **Forward Primer** | **Reverse Primer** |
| --- | --- | --- |
| Aggrecan | CTGAATGGGAGCCAGCCTAC | GATGTGGAAGGGACTTGCGA |
| Col2a1 | TTTGACGAGAAGGCTGGTGG | GGACCAATGGGACCAGAGAC |
| MMP13 | ACCATCCTGTGACTCTTGCG | TTCACCCACATCAGGCACTC |
| Piezo1 | CGACATTGACAACTGCACCG | GTCAGCCAGCTGTGGTACAT |
| ATF6 | CGAGGCTGGGTTCATAGACAT | GGCGAAGCGTAATACACCTG |
| XBP1s | GCTGAGTCCGCAGCAGGT | GGTCCAACTTGTCCAGAATGC |
| GAPDH | GAACATCATCCCTGCATCCA | GCCAGTGAGCTTCCCGTTCA |
