# Supplementary material for: Shengyu decoction ameliorates knee osteoarthritis by inhibiting endoplasmic reticulum stress via Piezo1 channels
Source: Front Pharmacol. 2025 Jul 14;16:1592818. doi: 10.3389/fphar.2025.1592818 (PMC12301362; doi:10.3389/fphar.2025.1592818)
Supplement: Supplementary file 5 [file Table2.docx]

**Supplementary Table 2**

| **RTmin** | **m/z** | **Type** | **Name** |
| --- | --- | --- | --- |
| 22.67 | 305.2465 | [M+H]+ | (5S,8R,9S,10S,13S,14S,17S)-17-hydroxy-10,13,17-trimethyl-2,4,5,6,7,8,9,11,12,14,15,16-dodecahydro-1H-cyclopenta[a]phenanthren-3-one |
| 22.44 | 303.2320 | [M+H]+ | (8R,9S,10R,13S,14S,17S)-17-hydroxy-10,13,17-trimethyl-2,6,7,8,9,11,12,14,15,16-decahydro-1H-cyclopenta[a]phenanthren-3-one |
| 20.92 | 621.3043 | [M+H]+ | 12b-O-[non-2Z,4E-dienoyl]-13_-isobutyroyloxy-7-oxo-5-ene-4b-phorbol |
| 20.64 | 526.2322 | [M+H]+ | NCGC00160137-01!2-[2-(3,4-Dimethoxy-phenyl)-ethyl]-6-(4-methoxy-phenyl)-2,3,6,7,12,12a-hexahydro-pyrazino[1',2':1,6]pyrido[3,4-b]indole-1,4-dione |
| 0.70 | 290.0837 | [M+H]+ | (2R,3R,4S,5R)-2-(6-amino-9H-purin-9-yl)-5-(hydroxymethyl)tetrahydrofuran-3,4-diol |
| 21.18 | 621.3007 | [M+H]+ | (2R,3R,4S,5R,6R)-2-[[7-[(2R,3R,4R,5S)-3,4-dihydroxy-5-(hydroxymethyl)oxolan-2-yl]oxy-2-ethenyl-2,4b,8,8-tetramethyl-4,4a,5,6,7,8a,9,10-octahydro-3H-phenanthren-3-yl]oxy]-6-methyloxane-3,4,5-triol |
| 0.72 | 337.1708 | [M+H]+ | (2R,3R,4S,5S,6R)-2-[(2E)-4-ethenyl-2,5-dimethylhexa-2,5-dienoxy]-6-(hydroxymethyl)oxane-3,4,5-triol |
| 20.90 | 603.2937 | [M+H]+ | (2S)-5,7-dihydroxy-2-(4-hydroxyphenyl)-6-[(2S,3R,4R,5S,6R)-3,4,5-trihydroxy-6-[[(2R,3R,4R,5R,6S)-3,4,5-trihydroxy-6-methyloxan-2-yl]oxymethyl]oxan-2-yl]-2,3-dihydrochromen-4-one |
| 16.71 | 1242.6150 | [M+H]+ | (2S,3R,4R,5R,6S)-2-{[(2R,3S,4S,5R,6R)-5-{[(2S,3R,4S,5R,6R)-3,5-dihydroxy-6-(hydroxymethyl)-4-{[(2S,3R,4S,5S,6R)-3,4,5-trihydroxy-6-(hydroxymethyl)oxan-2-yl]oxy}oxan-2-yl]oxy}-4-hydroxy-2-(hydroxymethyl)-6-[(1'S,2S,4'S,5S,7'S,8'R,9'S,13'R,16'S)-5,7',9',13'-tetramethyl-5-({[(2R,3R,4S,5S,6R)-3,4,5-trihydroxy-6-(hydroxymethyl)oxan-2-yl]oxy}methyl)-5'-oxaspiro[oxolane-2,6'-pentacyclo[10.8.0.0?,?.0?,?.0??,??]icosan]-18'-eneoxy]oxan-3-yl]oxy}-6-methyloxane-3,4,5-triol |
| 0.88 | 344.1339 | [M+Na]+ | (E)-3-[2-[(2S,3R,4S,5S,6R)-3,4,5-trihydroxy-6-(hydroxymethyl)oxan-2-yl]oxyphenyl]prop-2-enoic acid |
| 0.80 | 455.1873 | [M+H]+ | [(1S,3R,3aS,4S,8aR)-1-acetyloxy-3-hydroxy-6,8a-dimethyl-3-propan-2-yl-1,2,3a,4,5,8-hexahydroazulen-4-yl] 4-hydroxybenzoate |
| 0.84 | 334.0916 | [M+Na]+ | [3,4,5-trihydroxy-6-(hydroxymethyl)oxan-2-yl] 9-(hydroxymethyl)-2,2,6a,6b,9,12a-hexamethyl-10-[3,4,5-trihydroxy-6-[(3,4,5-trihydroxy-6-methyloxan-2-yl)oxymethyl]oxan-2-yl]oxy-1,3,4,5,6,6a,7,8,8a,10,11,12,13,14b-tetradecahydropicene-4a-carboxylate |
| 20.54 | 439.1262 | [M+H]+ | [3-hydroxy-4-[(2S,3R,4S,5S,6R)-3,4,5-trihydroxy-6-(hydroxymethyl)oxan-2-yl]oxyphenyl]methyl 3,4-dihydroxybenzoate |
| 0.59 | 537.1947 | [M+H]+ | [4-[3,4,5-trihydroxy-6-(hydroxymethyl)oxan-2-yl]oxyphenyl]methyl 3-acetyloxy-2-hydroxy-2-[(4-hydroxyphenyl)methyl]butanoate |
| 22.40 | 365.1355 | [M+Na]+ | [5-acetyloxy-3-(hydroxymethyl)-2-oxo-6-propan-2-ylcyclohex-3-en-1-yl] 3-methylbutanoate |
| 5.00 | 316.2108 | [M+H]+ | [5-hydroxy-3-(hydroxymethyl)-2-oxo-6-propan-2-ylcyclohex-3-en-1-yl] 3-methylpentanoate |
| 0.66 | 392.1046 | [M+H]+ | 1,7-bis(3,4-dihydroxyphenyl)heptan-3-yl acetate |
| 22.25 | 522.3555 | [M+H]+ | 1_18_1_lysophosphatidylcholine |
| 18.97 | 1285.6400 | [M+H]+ | 11-Oxomogroside V |
| 0.62 | 441.1978 | [M+H]+ | 19,20-epoxynovacine |
| 22.94 | 760.5820 | [M+H]+ | 1-palmitoyl-2-oleoyl-sn-glycero-3-phosphocholine; MALDI-TOFTOF; MS2; CE |
| 0.66 | 367.1589 | [M+H]+ | 2-(4-(2,3-dihydrobenzo[b][1,4]dioxin-6-yl)-3-methyl-1H-pyrazol-5-yl)-4-ethyl-5-methoxyphenol |
| 20.86 | 382.2711 | [M+H]+ | 2-[(2E)-3,7-dimethylocta-2,6-dienyl]-3-methoxy-5-(2-phenylethyl)phenol |
| 21.14 | 1115.2760 | [M+H]+ | 2-{[(17Z)-17-ethylidene-14-(1-hydroxyethyl)-27-(2-hydroxypropan-2-yl)-20,33-dimethyl-24,30,37,40-tetramethylidene-12,15,22,25,28,35,38-heptaoxo-19,32,42-trioxa-9-thia-3,13,16,23,26,29,36,39,44,45,46,47-dodecaazahexacyclo[39.2.1.1?,??.1??,??.1??,??.0?,?]heptatetraconta-1(43),2(7),3,5,8(47),10,18(46),20,31(45),33,41(44)-undecaen-4-yl]formamido}prop-2-enamide |
| 0.64 | 156.0419 | [M+H]+ | 2-amino-4-hydroxypyrimidine-5-carboxylic acid |
| 18.75 | 1138.6430 | [M+Na]+ | 3-[21-benzyl-3,24-bis(butan-2-yl)-6-(2-carboxyethyl)-7,10,28-trimethyl-2,5,8,11,14,20,23,26,29,32-decaoxo-9,30-bis(propan-2-yl)-25-oxa-1,4,7,10,13,19,22,28,31-nonaazatricyclo[31.4.0.0??,??]heptatriacontan-27-yl]propanoic acid |
| 0.64 | 465.9936 | [M+H]+ | 3-dihydrodiscorhabdin C (+2) |
| 0.64 | 184.0946 | [M+H]+ | 3-phenyllactic acid |
| 1.00 | 174.1121 | [M+H]+ | 4,5-dihydroxy-3-propylcyclopent-2-en-1-one |
| 2.26 | 188.0703 | [M+H]+ | 4-hydroxy-4-(pyridin-2-yl)butan-2-one |
| 9.74 | 668.3359 | [M+2H]2+ | 8-Deacetyl yunaconitine |
| 0.60 | 280.0919 | [M+H]+ | 9-Methoxy-2,2-dimethyl-2,6-dihydro-pyrano[3,2-c]quinolin-5-one |
| 22.27 | 438.2961 | [M+H]+ | Bullatine B |
| 0.84 | 312.1101 | [M+H]+ | C11H21NO7S; PlaSMA ID-1653 |
| 22.67 | 284.2945 | [M+H]+ | C18(Plasm)-18:1 PC |
| 22.83 | 383.3299 | [M+H]+ | Cholest-4,6-Dien-3-One |
| 21.26 | 383.3295 | [M+NH4]+ | Cholest-4,6-Dien-3-One |
| 0.98 | 316.1364 | [M+H]+ | CHOLESTANE |
| 22.96 | 369.3507 | [M+NH4]+ | Cholesterol; LC-APPI-QQ; MS2; CE |
| 17.53 | 373.2728 | [M+H]+ | cholic acid; CE0; BHQCQFFYRZLCQQ-OELDTZBJSA-N |
| 22.11 | 335.2791 | [M+H]+ | Dihydroxy (3Alpha,12Alpha)Pregnan-20-One |
| 0.68 | 203.0520 | [M+H]+ | Diphenylsulfoxide |
| 0.64 | 463.9964 | [M+H]+ | discorhabdin C isomer (+2) |
| 20.86 | 282.2787 | [M+H]+ | Dodemorph |
| 0.55 | 389.0082 | [M+Na]+ | dUDP |
| 22.94 | 338.3408 | [M+H]+ | Erucamide |
| 0.70 | 300.1051 | [M+H]+ | Erysovine |
| 17.95 | 387.1790 | [M+H]+ | Eudesmin |
| 17.95 | 404.2061 | [M+NH4]+ | eudesmin |
| 0.62 | 406.0371 | [M+H]+ | GALANTHAMINE HYDROBROMIDE |
| 21.88 | 437.3729 | [M+NH4]+ | gamma-sitosterol |
| 21.66 | 555.2935 | [M+H]+ | Ganoderic Acid G |
| 20.64 | 495.1895 | [M+H]+ | harpagoside |
| 22.31 | 327.3356 | [M+H]+ | henicosanoic acid |
| 22.37 | 327.2315 | [M+H]+ | hydroquinidine |
| 0.59 | 352.8972 | [M+H]+ | Hymenamide B |
| 17.53 | 817.5804 | [M+H]+ | hyocholic acid |
| 0.57 | 489.1037 | [M+H]+ | Irigenin, 7-Benzyl Ether |
| 22.07 | 581.3077 | [M+NH4]+ | Isocucurbitacin B |
| 0.64 | 257.1472 | [M+H]+ | Isocurcumenol |
| 17.47 | 274.2733 | [M+H]+ | ISOPALMITIC ACID |
| 22.40 | 163.0749 | [M+H]+ | Isosafrole |
| 0.70 | 162.1124 | [M+H]+ | L-Carnitine; CE0; PHIQHXFUZVPYII-ZCFIWIBFSA-N |
| 0.86 | 229.1544 | [M+H]+ | Leucylproline; PlaSMA ID-901 |
| 22.91 | 324.2887 | [M+H]+ | Linoleoyl Ethanolamide |
| 21.53 | 520.3400 | [M+NH4]+ | LPC 18:2; PlaSMA ID-2754 |
| 21.92 | 454.2925 | [M+H]+ | LPE 16:0; PlaSMA ID-2476 |
| 22.17 | 480.3090 | [M+H]+ | LPE 18:1; PlaSMA ID-2600 |
| 2.26 | 205.0967 | [M+NH4]+ | L-Tryptophan; CE0; QIVBCDIJIAJPQS-VIFPVBQESA-N |
| 18.81 | 657.2029 | [M+Na]+ | methyl 3-(4-(2-(2,3-dihydrobenzofuran-5-yl)ethoxy)-3-methoxyphenyl)-3-(2-(3,4-dihydroxyphenyl)-3,5,7-trihydroxy-4-oxo-4H-chromen-8-yl)propanoate |
| 0.60 | 221.0185 | [M+H]+ | methyl orsellinate |
| 21.30 | 1085.5490 | [M+H]+ | Methyl protodioscin |
| 21.08 | 463.1632 | [M+H]+ | Methylnissolin-3-O-glucoside |
| 19.60 | 387.1916 | [M+H]+ | MLS001140379-01!1-[2-(4-butyl-2-oxochromen-7-yl)oxyacetyl]piperidine-4-carboxamide |
| 21.08 | 494.3233 | [M+H]+ | MLS001333570-01!55297-96-6 |
| 0.66 | 411.1839 | [M+H]+ | MMV688467 |
| 0.88 | 366.1154 | [M+H]+ | MMV688798 |
| 0.70 | 274.0893 | [M+H]+ | Modafinil |
| 10.06 | 254.1744 | [M+H]+ | MPL-dm |
| 0.68 | 353.0921 | [M+H]+ | N4-Acetylsulfadimethoxine; LC-ESI-ITFT; MS2; CE |
| 22.38 | 572.3697 | [M+H]+ | NCGC00168955-03![3-hydroxy-1-oxo-1-(2,3,4,5,6-pentahydroxyhexoxy)decan-5-yl] 3,5-dihydroxydecanoate |
| 22.44 | 285.2208 | [M+H]+ | NCGC00169715-02_C20H30O2_Abieta-8(14),9(11),12-triene-7,18-diol |
| 21.68 | 313.2730 | [M+H]+ | NCGC00178646-02!2,4-dihydroxyheptadecyl acetate |
| 0.98 | 339.1541 | [M+H]+ | NCGC00381117-01!2-amino-4-hydroxy-5-[[1-(4-methoxy-6-oxopyran-2-yl)-3-methylbutyl]amino]-5-oxopentanoic acid |
| 14.38 | 1085.5080 | [M+H]+ | NCGC00384584-01_C51H82O23_(3beta,9xi,22S,25S)-26-(beta-D-Glucopyranosyloxy)-22,25-epoxyfurost-5-en-3-yl 6-deoxy-alpha-L-mannopyranosyl-(1->4)-beta-D-glucopyranosyl-(1->2)-beta-D-glucopyranoside |
| 16.90 | 1207.5720 | [M+H]+ | NCGC00384689-01_C57H90O27_ |
| 0.80 | 469.2040 | [M+NH4]+ | NCGC00384800-01_C21H34O10_ |
| 20.64 | 512.2157 | [M+NH4]+ | NCGC00384835-02![4-[3,4,5-trihydroxy-6-(hydroxymethyl)oxan-2-yl]oxyphenyl]methyl 2,3-dihydroxy-2-[(4-hydroxyphenyl)methyl]butanoate |
| 10.20 | 430.2414 | [M+H]+ | NCGC00385247-01_C21H32O8_(3S,3aS,6E,9S,10E,11aS)-3,6,10-Trimethyl-2-oxo-2,3,3a,4,5,8,9,11a-octahydrocyclodeca[b]furan-9-yl beta-D-glucopyranoside |
| 21.51 | 291.1950 | [M+H]+ | NCGC00385282-01_C18H28O4_(3E,5R,6E,8S,9E,13S,14R)-5-Hydroxy-8-methoxy-5,9,13,14-tetramethyloxacyclotetradeca-3,6,9-trien-2-one |
| 19.26 | 1283.6310 | [M+Na]+ | NCGC00385312-01_C60H98O29_alpha-L-Arabinopyranoside, (3beta,5xi,9xi,16alpha,18xi,22alpha)-22-(acetyloxy)-13,28-epoxy-16,23-dihydroxyoleanan-3-yl O-beta-D-glucopyranosyl-(1->2)-O-[O-beta-D-glucopyranosyl-(1->4)-O-beta-D-xylopyranosyl-(1->2)-beta-D-glucopyranosyl-(1->4)]- |
| 21.18 | 643.2825 | [M+Na]+ | NCGC00386007-01_C33H48O10_(2alpha,5alpha,9alpha,10beta,14beta)-2,5,9,10-Tetraacetoxytaxa-4(20),11-dien-14-yl 2-methylbutanoate |
| 21.12 | 579.2925 | [M+H]+ | NCGC00386015-01_C32H44O8_Estra-1,5-diene-3,11-dione, 17-[(1R,3E)-1-(acetyloxy)-5-hydroxy-1,5-dimethyl-2-oxo-3-hexen-1-yl]-2,16-dihydroxy-4,4,9,14-tetramethyl-, (9beta,10alpha,16alpha,17beta)- |
| 21.59 | 389.3376 | [M+H]+ | Nervonic Acid |
| 1.00 | 294.1548 | [M+H]+ | N-Fructosyl isoleucine; PlaSMA ID-1496 |
| 1.99 | 328.1389 | [M+H]+ | N-Fructosyl phenylalanine; PlaSMA ID-1767 |
| 0.84 | 276.0896 | [M+H]+ | norlichexanthone |
| 22.17 | 400.3418 | [M+Na]+ | Palmitoylcarnitine |
| 0.70 | 304.1003 | [M+H]+ | Pangamic Acid Sodium |
