# Supplementary material for: Shengyu decoction ameliorates knee osteoarthritis by inhibiting endoplasmic reticulum stress via Piezo1 channels
Source: Front Pharmacol. 2025 Jul 14;16:1592818. doi: 10.3389/fphar.2025.1592818 (PMC12301362; doi:10.3389/fphar.2025.1592818)
Supplement: Supplementary file 6 [file Table3.docx]

**Supplementary Table 3**

| **RTmin** | **m/z** | **Type** | **Name** |
| --- | --- | --- | --- |
| 20.53 | 453.3333 | [M-H]- | (1S,4S,5R,10S,13S,17S,19S,20R)-10-hydroxy-4,5,9,9,13,19,20-heptamethyl-24-oxahexacyclo[15.5.2.0?,??.0?,??.0?,??.0?,??]tetracos-15-en-23-one |
| 9.69 | 1161.5690 | [M-H]- | (3?,5?,9?,16?,18?,22?)-22-Acetoxy-16-hydroxy-13,28-epoxyoleanan-3-yl 6-deoxy-?-L-mannopyranosyl-(1->2)-?-D-galactopyranosyl-(1->3)-[?-D-glucopyranosyl-(1->2)]-?-D-glucopyran osiduronic acid |
| 17.15 | 451.2694 | [M-H]- | (E)-5-hydroxy-N-[3-[5-[3-[[(E)-5-hydroxy-3-methylpent-2-enoyl]amino]propyl]-3,6-dioxopiperazin-2-yl]propyl]-3-methylpent-2-enamide |
| 21.85 | 361.2381 | [M-H]- | (E)-8-(4-hydroxy-6-methoxy-7-methyl-3-oxo-1H-2-benzofuran-5-yl)-2,6-dimethyloct-6-enoic acid |
| 21.19 | 787.2008 | [M-H]- | [(2R,3R,4S,5R,6S)-2-(hydroxymethyl)-4,5,6-tris[(3,4,5-trihydroxybenzoyl)oxy]oxan-3-yl] 3,4,5-trihydroxybenzoate |
| 22.45 | 691.3892 | [M-H]- | [(2R,3R,4S,5S,6R)-3-acetyloxy-2-(acetyloxymethyl)-5-hexanoyloxy-6-[(2S,3R)-2,3,4-trihydroxybutoxy]oxan-4-yl] 14-hydroxytetradecanoate |
| 21.71 | 621.3058 | [M-H]- | [(2R,3S,4S,5R,6R)-6-[1,7-bis(4-hydroxyphenyl)-5-oxoheptan-3-yl]oxy-3,4,5-trihydroxyoxan-2-yl]methyl (E)-3-(4-hydroxyphenyl)prop-2-enoate |
| 21.23 | 739.2427 | [M-H]- | [(2R,3S,4S,5R,6S)-6-[(2S,3R,4R,5R,6S)-2-[5,7-dihydroxy-2-(4-hydroxyphenyl)-4-oxochromen-3-yl]oxy-4,5-dihydroxy-6-methyloxan-3-yl]oxy-3,4,5-trihydroxyoxan-2-yl]methyl (E)-3-(4-hydroxyphenyl)prop-2-enoate |
| 0.97 | 667.2284 | [M-H]- | [(2S,3R,4S,5R,6S)-6-[[(1aS,1bS,2S,5aR,6S,6aS)-1a-(hydroxymethyl)-2-[(2S,3R,4S,5S,6R)-3,4,5-trihydroxy-6-(hydroxymethyl)oxan-2-yl]oxy-2,5a,6,6a-tetrahydro-1bH-oxireno[5,6]cyclopenta[1,3-c]pyran-6-yl]oxy]-4,5-dihydroxy-2-methyloxan-3-yl] (E)-3-(4-methoxyphenyl)prop-2-enoate |
| 0.56 | 373.2367 | [M-H]- | 1,7-bis(3,4-dihydroxyphenyl)heptan-3-yl acetate |
| 22.49 | 547.2759 | [M-H]- | 1-[2,4,6-trihydroxy-3-[7-hydroxy-2-(4-hydroxyphenyl)-3,4-dihydro-2H-chromen-4-yl]phenyl]dodecan-1-one |
| 21.19 | 677.2480 | [M-H]- | 15-(carbamoylmethyl)-10,11,23-trihydroxy-18-(3-methyl-2-oxopentanamido)-9,14,17-trioxo-N-[(1Z)-prop-1-en-1-yl]-8,13,16-triazatetracyclo[18.3.1.0?,?.0?,??]tetracosa-1(23),2(7),3,5,20(24),21-hexaene-12-carboxamide |
| 22.12 | 468.1520 | [M-H]- | 2-[1-[4,5-dihydroxy-3-[(2S,3R,4S,5S,6R)-3,4,5-trihydroxy-6-(hydroxymethyl)oxan-2-yl]oxyoxan-2-yl]indol-3-yl]acetic acid |
| 21.31 | 681.2427 | [M-H]- | 2-[4-[(3S,3aR,6S,6aR)-6-[3-methoxy-4-[3,4,5-trihydroxy-6-(hydroxymethyl)oxan-2-yl]oxyphenyl]-1,3,3a,4,6,6a-hexahydrofuro[3,4-c]furan-3-yl]-2-methoxyphenoxy]-6-(hydroxymethyl)oxane-3,4,5-triol |
| 21.46 | 443.3491 | [M-H]- | 21-hydroxy-10-methoxy-1,4,14,19,19-pentamethyl-2,7,18-trioxapentacyclo[11.9.0.0?,??.0?,?.0??,??]docosa-3(11),4,9-triene-8,17-dione |
| 22.14 | 522.2841 | [M-H]- | 2-amino-3-[hydroxy-[2-hydroxy-3-[octadec-9-enoyl]oxypropoxy]phosphoryl]oxypropanoic acid |
| 0.56 | 347.2589 | [M-H]- | 2-hydroxy-6-pentadecylbenzoic acid |
| 0.61 | 1059.5050 | [M-H]- | 3-(3'-O-Malonyl)Glu(1-4)Glu-28-Glu Bayogenin (NMR) |
| 22.45 | 319.2276 | [M-H]- | 5-(1,2,4a,5-tetramethyl-7-oxo-3,4,8,8a-tetrahydro-2H-naphthalen-1-yl)-3-methylpentanoic acid |
| 22.04 | 608.3173 | [M-H]- | 6-(1H-indol-3-ylmethyl)-9-methyl-3,15-di(propan-2-yl)-1,4,7,10,13,16-hexazabicyclo[16.3.0]henicosane-2,5,8,11,14,17-hexone |
| 5.07 | 652.8983 | [M-H]- | 6-hydroxy-3-[3-hydroxy-4-[3,4,5-trihydroxy-6-[[3,4,5-trihydroxy-6-(hydroxymethyl)oxan-2-yl]oxymethyl]oxan-2-yl]oxyphenyl]-5,7-dimethoxychromen-4-one |
| 20.32 | 359.2264 | [M-H]- | 7-O-Methylrosmanol |
| 22.45 | 301.2169 | [M-H2O-H]- | abietic acid |
| 19.85 | 475.3531 | [M-H]- | arthrobactin |
| 22.35 | 325.2383 | [M-H]- | Avocadyne Acetate |
| 20.11 | 593.2728 | [M-H]- | C27H46O14; PlaSMA ID-1644 |
| 0.69 | 765.4432 | [M-H]- | Cauloside C (3-Glu(1-2)Ara Hederagenin) (NMR) |
| 10.44 | 829.3716 | [M-2H]2- | Chukrasin Methyl Ether |
| 20.17 | 483.3452 | [M-H]- | Dihydrogedunin |
| 10.60 | 806.3427 | [M-2H]2- | Docetaxel |
| 20.59 | 265.1477 | [M-H]- | Dodecyl sulfate |
| 20.85 | 614.3300 | [M-H]- | Hexosyl LPE 16:0; PlaSMA ID-1697 |
| 21.07 | 638.3298 | [M-H]- | Hexosyl LPE 18:2; PlaSMA ID-1765 |
| 21.95 | 707.2609 | [M-H]- | Icariin; LC-ESI-ITTOF; MS2; [(M+CH3COOH)-H]- |
| 11.06 | 639.1945 | [M-H]- | isosakuranetin-7-O-neohesperidoside; PlaSMA ID-1771 |
| 1.01 | 723.1873 | [M-H]- | Lepraric acid |
| 21.36 | 279.2320 | [M-H]- | Linoleic acid |
| 22.03 | 540.3306 | [M-H]- | LPC 16:0; PlaSMA ID-1486 |
| 22.26 | 566.3467 | [M-H]- | LPC 18:1; PlaSMA ID-1563 |
| 21.54 | 564.3295 | [M-H]- | LPC 18:2; PlaSMA ID-1554 |
| 21.69 | 452.2776 | [M-H]- | LPE 16:0; PlaSMA ID-1223 |
| 16.21 | 624.2953 | [M-H]- | LTC4; LC-ESI-QIT; MS2; CE |
| 21.17 | 755.2626 | [M-H]- | methyl (1S,4aS,7aS)-7-(hydroxymethyl)-1-[(2S,3R,4S,5S,6R)-3,4,5-trihydroxy-6-[[(2R,3R,4S,5S,6R)-3,4,5-trihydroxy-6-[[(E)-3-(4-hydroxy-3,5-dimethoxyphenyl)prop-2-enoyl]oxymethyl]oxan-2-yl]oxymethyl]oxan-2-yl]oxy-1,4a,5,7a-tetrahydrocyclopenta[c]pyran-4-carboxylate |
| 21.11 | 695.2155 | [M-H]- | methyl (1S,4aS,7aS)-7-(hydroxymethyl)-1-[(2S,3R,4S,5S,6R)-3,4,5-trihydroxy-6-[[(2R,3R,4S,5S,6R)-3,4,5-trihydroxy-6-[[(E)-3-(4-hydroxyphenyl)prop-2-enoyl]oxymethyl]oxan-2-yl]oxymethyl]oxan-2-yl]oxy-1,4a,5,7a-tetrahydrocyclopenta[c]pyran-4-carboxylate |
| 0.56 | 437.1556 | [M-H]- | methyl 2-[[4-hydroxy-3-(3-methylbut-2-enyl)phenyl]methyl]-3-(4-hydroxyphenyl)-4-methoxy-5-oxofuran-2-carboxylate |
| 21.54 | 561.3313 | [M-H]- | MGMG 18:2; PlaSMA ID-1541 |
| 0.81 | 579.2370 | [M-H]- | naringin |
| 21.95 | 658.3309 | [M-H]- | NCGC00165733-02!(3S,6S,9S,12R)-3-[(2S)-butan-2-yl]-6-[(1-methoxyindol-3-yl)methyl]-9-(6-oxooctyl)-1,4,7,10-tetrazabicyclo[10.4.0]hexadecane-2,5,8,11-tetrone |
| 0.81 | 749.1892 | [M-H]- | NCGC00179806-02![4,5-diacetyloxy-6-[7-hydroxy-5-methoxy-4-oxo-2-[4-[3,4,5-trihydroxy-6-(hydroxymethyl)oxan-2-yl]oxyphenyl]chromen-6-yl]oxan-3-yl] acetate |
| 0.99 | 749.2025 | [M-H]- | NCGC00180378-02! |
| 21.09 | 743.3486 | [M-H]- | NCGC00347423-02!3-[(3S,5S,8R,10S,13R,14S,17R)-3-[4,5-dihydroxy-6-(hydroxymethyl)-3-[3,4,5-trihydroxy-6-(hydroxymethyl)oxan-2-yl]oxyoxan-2-yl]oxy-14-hydroxy-10,13-dimethyl-1,2,3,4,5,6,7,8,9,11,12,15,16,17-tetradecahydrocyclopenta[a]phenanthren-17-yl]-2H-furan-5-one |
| 21.44 | 700.3987 | [M-H]- | NCGC00347652-02_C35H53N5O7_12-Benzyl-3-isobutyl-6,9-diisopropyl-5,8-dimethyldodecahydro-1H,3H-pyrrolo[1,2-q][1,4,7,10,13,17]oxapentaazacyclononadecine-1,4,7,10,13,17(14H)-hexone |
| 10.72 | 664.2960 | [M-H]- | NCGC00373238-02_C33H47NO13_Delvocid |
| 20.85 | 571.2906 | [M-H]- | NCGC00380815-01_C31H42O7_5-Heptenoic acid, 2-[(4alpha,5alpha,6beta,8alpha,9beta,13alpha,14beta,16beta,17E)-16-(acetyloxy)-6-hydroxy-4,8,10,14-tetramethyl-3,7-dioxogon-1-en-17-ylidene]-6-methyl-, (2E)- |
| 16.41 | 695.2555 | [M-H]- | NCGC00380896-01_C32H42O14_ |
| 21.71 | 567.3162 | [M-H]- | NCGC00381203-01_C31H46N4O7_2H-Pyrido[1,2-a][1,4,7,10]tetraazacyclododecine-1,4,7,10(3H,12H)-tetrone, 3-(7,8-dihydroxy-6-oxooctyl)octahydro-9-(1-methylpropyl)-6-(phenylmethyl)- |
| 22.37 | 606.3322 | [M-H]- | NCGC00381238-01_C31H47NO8_2-[(2E,5E,7E,11E)-10-Hydroxy-3,7,9,11-tetramethyl-2,5,7,11-tridecatetraen-1-yl]-5,6-dimethoxy-3-methyl-4-pyridinyl 6-deoxy-alpha-L-mannopyranoside |
| 22.03 | 1135.5900 | [M-H]- | NCGC00381293-01_C55H92O24_Hexopyranoside, (3beta,9xi,11alpha,12beta,14beta,20S)-8,11,12,14,20-pentahydroxypregn-5-en-3-yl O-hexopyranosyl-(1->4)-O--6-deoxy-3-O-methylhexopyranosyl-(1->4)-O--2,6-dideoxy-3-O-methylhexopyranosyl-(1->4)-O--2,6-dideoxy-3-O-methylhexopyranosyl-(1->4)-2,6-dideoxy-3-O-methyl- |
| 22.24 | 683.2632 | [M-H]- | NCGC00385666-01!(1R,2S)-7-hydroxy-1-(4-hydroxy-3,5-dimethoxyphenyl)-2-N,3-N-bis[2-(4-hydroxyphenyl)ethyl]-6,8-dimethoxy-1,2-dihydronaphthalene-2,3-dicarboxamide |
| 0.99 | 292.1401 | [M-H]- | N-Fructosyl isoleucine; PlaSMA ID-544 |
| 22.74 | 398.3275 | [M-H]- | Palmitoylcarnitine cation |
| 22.45 | 840.5777 | [M-H]- | PC(16:0e/11,12-EpETE) |
| 22.35 | 864.5788 | [M-H]- | PC(16:1e/17-HDoHE) |
| 1.31 | 730.5007 | [M-H]- | PE(16:0/9-HODE) |
| 21.65 | 603.0085 | [M-H]- | PFAP-N_PFSE_disubstituted; C12H19F10N2O8PS2 |
| 0.58 | 611.9504 | [M-H]- | PFCA-diether_Hsubstituted; C11H2F21O5 |
| 0.58 | 681.9397 | [M-H]- | PFCA-diether_Hsubstituted; C11H2F23O7 |
| 0.69 | 511.9579 | [M-H]- | PFCA-diether_Hsubstituted; C9H2F17O5 |
| 22.45 | 706.9131 | [M-H]- | PFCA-ether; C11HF21O11 |
| 17.46 | 772.9088 | [M-2H]2- | PFCA-ether; C12HF23O12 |
| 16.86 | 1186.9160 | [M-H]- | PFCA-unsaturated; C25HF45O2 |
| 17.48 | 1190.9130 | [M-H]- | PFCA-unsaturated_ether; C24HF45O3 |
| 14.64 | 758.9125 | [M-2H]2- | PFSA-H; C14H2F26O4S |
| 5.07 | 656.8880 | [M-H]- | PFSA-pentafluorosulfide; C9HF23O3S2 |
| 22.43 | 798.5663 | [M-H]- | Phosphatidylcholine 15 |
| 22.43 | 800.5820 | [M-2H]2- | Phosphatidylcholine 17 |
| 21.71 | 512.3368 | [M-H]- | Phosphatidylcholine lyso 15 |
| 22.37 | 524.3364 | [M-H]- | Phosphatidylcholine lyso 16 |
| 22.59 | 550.3516 | [M-H]- | Phosphatidylcholine lyso 18 |
| 22.82 | 578.3817 | [M-H]- | Phosphatidylcholine lyso 20 |
| 1.33 | 726.5064 | [M-H]- | Phosphatidylethanolamine 17 |
| 22.82 | 724.5273 | [M-H]- | Phosphatidylethanolamine alkenyl 16 |
| 22.70 | 726.5416 | [M-H]- | Phosphatidylethanolamine alkenyl 18 |
| 21.50 | 452.2778 | [M-H]- | Phosphatidylethanolamine lyso 16 |
| 18.35 | 476.2781 | [M-H]- | Phosphatidylethanolamine lyso 18 |
| 20.97 | 502.2948 | [M-H]- | Phosphatidylethanolamine lyso 20 |
| 22.26 | 436.2835 | [M-H]- | Phosphatidylethanolamine lyso alkenyl 16 |
| 22.49 | 462.2999 | [M-H]- | Phosphatidylethanolamine lyso alkenyl 18 |
| 22.76 | 885.5486 | [M-H]- | Phosphatidylinositol 18 |
| 21.67 | 571.2872 | [M-H]- | Phosphatidylinositol lyso 16 |
| 21.17 | 619.2878 | [M-H]- | Phosphatidylinositol lyso 20 |
| 22.45 | 814.5591 | [M-H]- | Phosphatidylserine 18 |
| 0.81 | 468.1334 | [M-H]- | Rhizocarpic acid |
| 22.70 | 414.2635 | [M-H]- | Salmeterol |
| 0.63 | 750.4434 | [M-H]- | Solanidine base + O-Hex-dHex; PlaSMA ID-1982 |
| 0.97 | 785.1888 | [M-H]- | Flavone base + 4O, C-Hex-FeruloylHex; PlaSMA ID-2035 |
| 21.85 | 317.2115 | [M-H]- | 12-HEPE; LC-ESI-QIT; MS2; CE |
| 22.97 | 610.4493 | [M-H]- | Phosphatidylcholine lyso 22 |
| 14.39 | 1029.5760 | [M-H]- | taurocholic acid |
