# Supplementary material for: Shengyu decoction ameliorates knee osteoarthritis by inhibiting endoplasmic reticulum stress via Piezo1 channels
Source: Front Pharmacol. 2025 Jul 14;16:1592818. doi: 10.3389/fphar.2025.1592818 (PMC12301362; doi:10.3389/fphar.2025.1592818)
Supplement: Supplementary file 7 [file Table4.docx]

**Supplementary Table 4**. The molar concentrations of the four compounds of SYD

*in vitro*

|  | Saikosaponin C | Saikosaponin D | Quercetin | Naringin |
| --- | --- | --- | --- | --- |
| Content (mg/g) | 0.33542 | 0.09929 | 0.27153 | 0.2117 |
| MW | 927.12 | 780.98 | 302.24 | 580.53 |
| SYD-L | 0.22612 μM | 0.07946 μM | 0.56149 μM | 0.227915 μM |
| SYD-M | 0.45224 μM | 0.15892 μM | 1.12298 μM | 0.45583 μM |
| SYD-H | 0.90448 μM | 0.31784 μM | 2.24596 μM | 0.91166 μM |
